# Emerging Frontiers in Robotic Upper-Limb Prostheses: Mechanisms, Materials, Tactile Sensors and Machine Learning-Based EMG Control: A Comprehensive Review

**DOI:** 10.3390/s25133892

**Published:** 2025-06-22

**Authors:** Beibit Abdikenov, Darkhan Zholtayev, Kanat Suleimenov, Nazgul Assan, Kassymbek Ozhikenov, Aiman Ozhikenova, Nurbek Nadirov, Akim Kapsalyamov

**Affiliations:** 1 Science and Innovation Center “Artificial Intelligence”, Astana IT University, Astana 010000, Kazakhstan; beibit.abdikenov@astanait.edu.kz (B.A.); d.zholtayev@reliveres.com (D.Z.); 2 ReLive Research, Astana 010000, Kazakhstan; kanat.suleimenov@ut.ee (K.S.); n.assan@reliveres.com (N.A.); n.nadirov@reliveres.com (N.N.); 3 Department of Information Technology and Entrepreneurship, Narva College, University of Tartu, 20307 Narva, Estonia; 4 Institute of Automation and Information Technologies, Satbayev University, Almaty 050000, Kazakhstan; k.ozhikenov@satbayev.university (K.O.); a.ozhikenova@satbayev.university (A.O.); 5 Department of Orthopedics, Mother and Child Health Center, Corporate Fund University Medical Center, Astana 010000, Kazakhstan; 6 Faculty of Engineering and Mathematics, Hochschule Bielefeld, 33619 Bielefeld, Germany

**Keywords:** control, EMG signal processing, machine learning, prosthetic materials, robotic hand prosthesis, tactile sensing

## Abstract

Hands are central to nearly every aspect of daily life, so losing an upper limb due to amputation can severely affect a person’s independence. Robotic prostheses offer a promising solution by mimicking many of the functions of a natural arm, leading to an increasing need for advanced prosthetic designs. However, developing an effective robotic hand prosthesis is far from straightforward. It involves several critical steps, including creating accurate models, choosing materials that balance biocompatibility with durability, integrating electronic and sensory components, and perfecting control systems before final production. A key factor in ensuring smooth, natural movements lies in the method of control. One popular approach is to use electromyography (EMG), which relies on electrical signals from the user’s remaining muscle activity to direct the prosthesis. By decoding these signals, we can predict the intended hand and arm motions and translate them into real-time actions. Recent strides in machine learning have made EMG-based control more adaptable, offering users a more intuitive experience. Alongside this, researchers are exploring tactile sensors for enhanced feedback, materials resilient in harsh conditions, and mechanical designs that better replicate the intricacies of a biological limb. This review brings together these advancements, focusing on emerging trends and future directions in robotic upper-limb prosthesis development.

## 1. Introduction

The vast majority of activities of daily living are performed with hands. And thus, the loss of hands due to amputation causes significant issues in a person’s independence. There are various causes of limb loss, such as trauma (mostly traffic collisions, workplace injuries, post-war zones, and occasionally natural disasters), vascular diseases (different types of diabetes, gangrene, infection, and peripheral artery diseases), cancer (bone cancer or soft tissue sarcomas), and birth-in-the-way conditions (some people are born with missing or deformed limbs due to genetic or development issues) [1,2,3]. Moreover, recent epidemiological data show that there is a steady increase in upper-limb amputations globally, increasing the demand for advanced prosthetic solutions [4].

The development of robotic prostheses has allowed many patients to regain partial independence and carry out some of their daily tasks on their own. Despite these advancements, most prostheses still need further improvements to achieve seamless interaction with both the user and the environment. A key challenge lies in accurately interpreting the user’s intent through electromyography (EMG) sensors, which capture muscle signals that must be decoded for effective control. Numerous research studies indicate that high-density EMG arrays can capture more nuanced muscle activity that improve the accuracy of intent detection [5]. Recently, machine learning has emerged as a powerful tool in processing raw, multi-channel EMG data and translating it into precise movement commands. These commands, in turn, must be transmitted to the prosthetic hardware to execute the desired motions with sufficient torque. Various algorithms—ranging from conventional approaches like support vector machines to deep learning networks—have demonstrated significant promise in decoding complex muscle signals [6]. Another critical factor in prosthesis design is biocompatibility. The prosthetic interface should integrate with the residual limb without causing skin irritation or other injuries, especially over prolonged periods of use. Consequently, materials must be carefully selected to ensure both structural integrity and user comfort, thus enhancing the overall experience and functionality of the device. Innovations in hypoallergenic and thermally adaptive materials show potential in reducing sweat build-up and skin irritation, contributing to longer daily wear times [7]. Various reviews were introduced in the field focusing only on some particular aspect of the prosthetics design such as control method, linkage-based mechanism design, biomedical polymers and the others [8,9,10,11,12]. However, they still lack a unified synthesis that explains how these components interact and together shape everyday reliability and user comfort. Further attention to which material-sensor types can ensure comfortable and durable daily use is still lacking.

Adverse weather conditions, such as extreme cold, snow, or rain, can pose significant challenges for prosthesis users. These conditions pose challenges not only for the mechanical structure of the prosthesis but also for its integrated sensors. Low temperatures, for example, can reduce the flexibility of certain materials and may lead to brittleness over time. Similarly, high humidity and airborne particles such as dust can disrupt the consistency and accuracy of sensor readings. Tactile sensing elements are especially vulnerable to such interference, as their performance can degrade when exposed to harsh environments. In colder climates, fluctuations in temperature can also influence the electrical behavior of sensor components, potentially resulting in unstable feedback during use. In this review, we investigate the key internal characteristics of contemporary prostheses, focusing on the machine learning (ML) methods employed to decode EMG signals, their relative effectiveness, and the control strategies implemented. We also examine the materials and tactile sensors that can be integrated into prosthetic hands. The goal of this review is to provide evidence-based recommendations on the features that modern prostheses should include—particularly with regard to weather resilience, sensor performance, and overall functionality. In doing so, we highlight the current state-of-the-art mechanisms and internal structures that aim to enhance usability and reliability for human subjects in diverse environmental conditions. To the best of the authors’ knowledge, no previous review has comprehensively examined these characteristics from all of the aforementioned perspectives.

**Figure 1:**
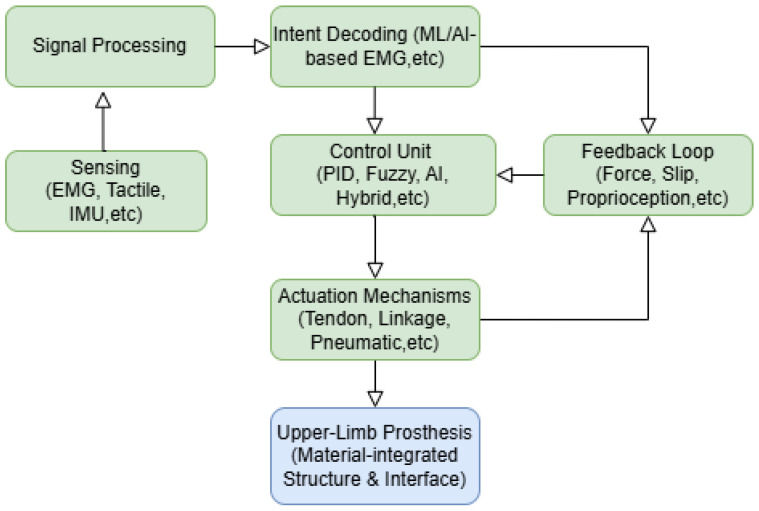
Overall schematic of key functional modules in upper-limb prosthetic systems, illustrating the flow from sensing and signal processing to control, materials integration, and final actuation.

The manuscript is structured as follows. Section 2 presents the methodology, detailing the systematic literature search conducted across Scopus, IEEE Xplore, ScienceDirect, and PubMed, including inclusion and exclusion criteria. Section 3 discusses the state-of-the-art ML methods employed for upper-limb prostheses, focusing on EMG signal processing, classification methods, and adaptive learning strategies. Section 4 explores control strategies for robotic prosthetic limbs, including traditional proportional derivative (PD)/ proportional integral derivative (PID) control, fuzzy logic approaches, and artificial intelligence (AI)-based adaptive control. Section 5 examines various transmission mechanisms, such as tendon-driven, linkage-based, and pneumatic actuation systems, comparing their advantages and limitations. Section 6 reviews tactile sensing technologies, emphasizing the role of capacitive, piezoresistive, and piezoelectric sensors in improving user interaction and prosthesis feedback. Section 7 provides a comprehensive overview of the type of materials that are applied in prosthetic devices, and the final Section 8 and Section 9 provide a discussion and conclusion, respectively, summarizing the key findings, identifying challenges, and highlighting potential future directions in robotic upper-limb prosthesis research. Figure 1 presents an overall schematic that outlines the interconnections between the major technical modules discussed in this review. The diagram illustrates how sensing, signal processing, control strategies, material selection, and feedback mechanisms integrate to form a functional upper-limb prosthetic system. This review treats the prosthesis as one joined-up system, so every later section links back to Figure 1 to show how control, sensors, mechanics and materials all work together.

## 2. Methods

A comprehensive literature search was conducted across four major databases—Scopus, IEEE Xplore, ScienceDirect, and PubMed—to identify the peer-reviewed studies on upper-limb prostheses and robotic hand mechanisms. The search was restricted to articles published in English, and initially no time range limit was imposed (except for the mechanism configuration whose search period of 2018–2025 was specified).

### 2.1. Search Strategy

The following keywords and phrases were used, often in Boolean combinations (e.g., “mechanism” AND “upper limb prosthesis”, “robotic hand” OR “prosthetic hand”):

- Core concepts: mechanism, driving, actuation, driven, control, sensor, EMG-based, force feedback, adaptive, bio-inspired, soft robotics, compliant, design, kinematics, biomechanics, ergonomics, rehabilitation, assistive technology, neuroprosthetics, materials, lightweight, 3D printing, soft materials.
- Target applications: upper-limb prosthesis, robotic hand, prosthetic hand, robotic finger, prosthetic finger.

Search queries typically combined at least one core concept term (e.g., “control” OR “actuation”) with at least one target application term (e.g., “upper limb prosthesis” OR “robotic hand”).

### 2.2. Inclusion and Exclusion Criteria

Studies were considered eligible if they met the following criteria:

- Focused on the design, control, or material selection of upper-limb prostheses or robotic hands/fingers.
- Reported on experimental or numerical analyses relating to biomechanical performance, actuation mechanisms, sensor integration, or EMG-based control strategies.
- Written in English.

The following studies were excluded:

- Review papers, meta-analyses, systematic reviews, short letters, unpublished materials, preprints, or surveys.
- Outside the scope of upper-limb prostheses (e.g., focusing solely on lower-limb devices or general robotics without clear application to prosthetics).
- Non-peer-reviewed or missing crucial methodological information.

### 2.3. Screening and Selection Process

All titles and abstracts retrieved were first screened to remove obviously irrelevant articles (e.g., unrelated to prosthetics, focusing on unrelated medical conditions, or addressing non-robotic devices). Next, duplicates were automatically removed using reference management software, followed by a manual check to ensure accuracy. Full-text articles of the remaining records were then assessed against the inclusion/exclusion criteria. To ensure the quality of the studies included, articles that lacked peer review or clear methods were excluded and the works that had experimental results or comparisons were prioritized to be included. While a formal risk-of-bias tool was not directly used, bias was minimized by cross-verifying findings across multiple high-quality studies and databases.

### 2.4. Data Extraction

For each included study, relevant information—such as actuation mechanism, control approach (including EMG signal processing and machine learning techniques), sensor integration (tactile and force feedback), and material usage—was extracted and tabulated. Particular attention was given to experimental validation, reported performance metrics, robustness under real-world conditions, and the presence of feedback loops for adaptive control.

### 2.5. Data Synthesis and Analysis

The extracted data were qualitatively synthesized to identify recurring themes and technological trends. Categories included the following:

1. State-of-the-art ML algorithms for myoelectric control: including feature extraction and classification techniques for EMG signal processing (e.g., convolutional neural networks (CNNs), transfer learning, incremental learning, and domain adaptation).
2. Transmission mechanisms (e.g., tendon-driven, linkage-based, and pneumatic).
3. Control strategies (e.g., classical control, fuzzy logic, machine learning, and deep learning).
4. Sensor technologies (e.g., tactile, force/torque, and EMG).
5. Materials (e.g., structural and socket materials, and soft interface components).

These categories served as a basis for organizing and discussing the state of the art in upper-limb prosthetic research, highlighting gaps and potential future directions.

## 3. State-of-the-Art Machine Learning Methods for Sensor-to-Command Conversion in Upper Limb Robotic Prostheses

### 3.1. Foundations of EMG-Based Prosthetic Control

Over the past few decades, prosthetic technology has advanced dramatically through the integration of EMG signals and ML algorithms [9]. By decoding the electrical activity generated by muscle contractions, myoelectric interfaces offer amputees an intuitive, user-friendly control scheme that can restore significant function and independence [9,13]. Designing robust acquisition systems that faithfully capture and preprocess these signals—often via empirical mode decomposition, band-pass and notch filtering—is essential for reliable classification. Feature extraction then translates raw EMG into discriminative inputs (e.g., time-domain metrics such as mean absolute value and root mean square, or time–frequency measures via wavelet transforms) that underpin accurate pattern recognition [14].

**Figure 2:**
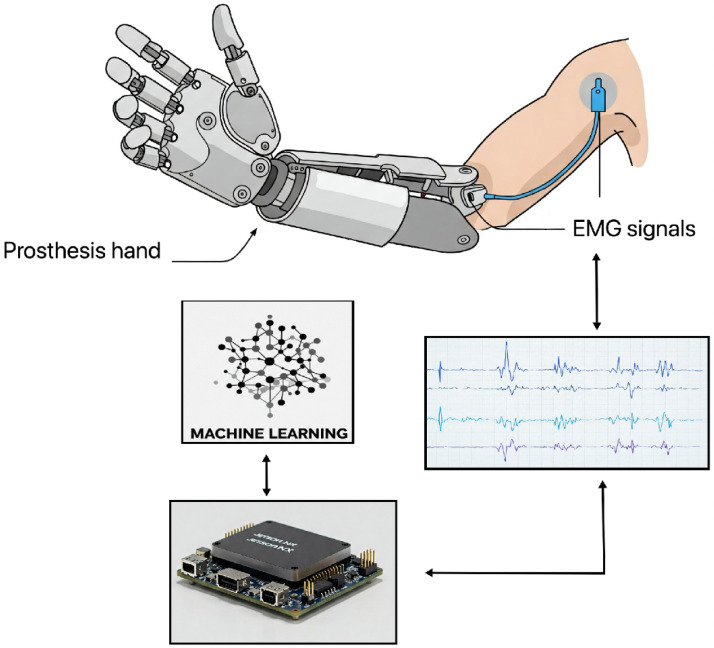
EMG-based prosthesis control using machine learning methods.

The integration of EMG signals into human–machine interfaces (HMIs) has greatly improved prostheses, helping people with limb loss regain function and independence. These interfaces use the electrical signals produced by muscle contractions (see Figure 2) as a reliable control input for artificial limbs and other assistive devices. Innovations in ML and deep learning (DL) algorithms enable an exact detection of human movement patterns as prosthesis design gets ever more sophisticated, hence improving both usability and functionality.

Modern research highlights that accurate pattern recognition (PR) is primarily dependent on high-quality signal capture, noise reduction, and feature extraction. Common preprocessing techniques include empirical mode decomposition, band-pass, and notch filtering, each of which helps to improve signal quality and reduce noise. Important for accurate classification, feature extraction usually consists of time domain (TD) and time–frequency domain (TFD) measurements, including mean absolute value (MAV), root mean square (RMS), and wavelet transforms. Particularly when coupled with transfer learning techniques, certain machine learning models ((SVM), (kNN), (ANN)) and sophisticated networks like CNNs have shown outstanding performance.

EMG signal analysis still faces challenges like noise contamination, variability connected with electrode location, and computing overhead in high-dimensional feature extraction even with constant improvement. Further complicating the generalizing of ML models are the lack of consistent data collecting techniques and inter-subject heterogeneity. Resilient preprocessing, adaptive algorithms, and economically feasible technologies [9] are therefore more important since balancing system performance and stability in multi-degree-of-freedom prosthesis remains a continuous challenge.

### 3.2. Advanced Machine Learning Strategies and Domain Adaptation

Modern developments in EMG-based control systems now also reach upper-limb prosthetic control. For example, using EMG-based classification and regression, dexterous robotic telemanipulation systems let individuals naturally manipulate robotic instruments. Evaluating several machine learning models and feature extraction techniques, the work by Godoy et al. highlighted Temporal Multi-Channel Transformers for real-time control in telemanipulation applications. This shows the great potential of shared control systems in challenging motor activities [15].

Similarly, incremental learning techniques have surfaced to satisfy long-term needs in myoelectric control. Context-informed Incremental Learning (CIIL), an adaptive framework including environmental feedback to maintain system robustness across time, was first presented by Campbell et al. CIIL greatly lowers recalibration requirements by reducing electrode displacement and user fatigue effects, hence outperforming state-of-the-art unsupervised adaptation methods [16]. Apart from direct control, EMG has helped to classify movement patterns in clinical diagnosis and tailored treatment. Zhang et al., for instance, introduced a hybrid neural network with transfer learning for cross-subject gait analysis, therefore addressing the generalization constraints of conventional models [17]. With possible uses in senior injury prevention and rehabilitation, their technique was quite good in identifying fall-related gait impairments. For exoskeleton control, meantime, gesture detection depends critically on multimodal sensing and feature extraction methods. By means of normalized EMG signals and support vector machines, Hazar and Ertuggrul created a real-time hand exoskeleton system capable of discriminating 32 gestures with 99.9% accuracy [18].

In myoelectric prosthesis, explainable artificial intelligence (XAI) has given EMG signal decoding a new level. Gozzi et al. (2022) used XAI to analyze black-box ML models for hand gesture classification, hence mapping muscle activity and exposing electrode placement anomalies [19]. In a similar attempt, Jarrah et al. (2022) addressed noisy EMG signals from transhumeral amputees using optimal filters including Wiener and Hampel, hence producing considerable gains in classification accuracy [20]. Real-time control and continuous motion prediction have been other subjects of study. In a viscous force field, Quesada et al. (2025) examined how muscle selection and feature extraction affect joint torque prediction, hence stressing the trade-offs between latency and accuracy [21]. To maximize trajectory estimation under fatigue [22], Davarinia and Maleki (2024) also presented a bimodal technique combining EMG with Steady-State Visual Evoked Potential (SSVEP) signals. Transient EMG classifiers are another method that emphasizes the transient period of muscle contraction for more accurate cross-subject wrist and hand movement detection [23].

### 3.3. Low-Cost, User-Oriented Advances in EMG-Based Upper-Limb Prosthesis Control

The Smart Demonstration Unit (SDU) by Potenza et al. (2024) accelerates calibration and training processes for underactuated robotic prosthesis access efforts [24]. An additional emphasis has been strong performance in daily surroundings. Using fuzzy logic to create a noise-tolerant dual multi-classifier system, Trajdos (2025) produced great dependability for daily prosthesis use [25]. Li et al. (2023) applied a regression-based simultaneous and proportional control technique for two degrees of freedom (DOFs) in myoelectric prostheses, enhancing task efficiency and user experience [26]. Further investigation methods to overcome the constraints of EMG are alternative sensing techniques. Based on the magnetic detection of muscle contractions, Prakash et al. (2022) presented Hall Myography (HMG), a reasonably affordable approach for prosthesis control in environments with limited resources [27].

System robustness still depends critically on daily EMG fluctuation. While López et al. (2024) combined CNN and long short-term memory (LSTM) architectures to reject errant labels, boosting classification accuracy [28], Lee et al. (2024) addressed this issue by proposing a recursive domain-adversarial neural network (DANN) framework with semi-supervised strategies [29]. Context-dependent signal interpretation further refines the classification results by interpreting identical EMG signals differently depending on task context [30]. Cutipa-Puma et al. (2023) included EEG signals and haptic feedback in a low-cost 3D-printed hand prosthesis [31]. For instance, Kim et al. [32] demonstrated how EEG feedback can be used to evaluate satisfaction levels and adjust control strategies accordingly. Such approaches highlight the importance of including neurophysiological metrics for assessing usability and performance alignment from the user’s perspective.

Furthermore, first and foremost in research has been recalibration efficiency. Calado et al. (2024) used geometric algebra-based classifiers for myoelectric pattern detection, hence reducing disturbances from electrode displacement and user tiredness [33]. By assessing generic EMG-torque models across several joints, Wang et al. (2024) simplified calibration and showed that generic models could be efficiently reused with minimum performance loss [34]. In multi-degree-of-freedom (multi-DOF) systems, the optimization of pattern recognition is especially crucial. By means of an optimal PR framework for the Hannes prosthesis, Marinelli et al. (2024) lowered sensor load and computational cost while preserving strong performance [35]. By using an amplitude-based window recognition algorithm on Arduino hardware, Unanyan and Belov (2021) presented low-cost alternatives that effectively mimic functionality without costly components [36].

Complementing these technical developments, the research also tackles usability frameworks. By assessing learnability, efficiency, and user satisfaction [37], Park et al. (2024) presented the Human Performance Model for Upper-Limb Prosthetic Devices (HPM-UP) For different patient demands, Fink and Diamondz (2023) underlined the need of balancing cost, usability, and functionality [38]. Further development in motor image (MI) decoding uses ML methods for exact prosthesis control. While Shi et al. (2022) addressed variability by a multi-task dual-stream supervised domain adaptation (MDSDA) network [39], Ma et al. (2022) used a time-distributed attention network for EEG-based MI decoding [40]. Control system design also advances through new actuation and modeling: Cho et al. (2022) introduced twisted string actuators for multi-finger control [41], and Rajapriya et al. (2021) adopted wavelet bispectrum features to handle variability in forearm orientation and contraction force [42]. Effective prosthetic function depends on sensory feedback; this was shown by Cha et al. (2022) with a proprioceptive haptic feedback device [43] and Li and Brown (2023) with dual-modality vibration and squeeze cues [44]. Table 1 presents a thorough comparison of several machine learning methods applied for EMG sensor-based solutions, stressing their performance over many evaluation criteria.

Adaptive modeling and reinforcement learning help to sustain efforts on personalization. Personalized EMG-driven musculoskeletal models [46] were presented by Berman et al. (2024) using a reinforcement learning framework Emphasizing human-centered design to increase user acceptance [48], Liu et al. (2024) evaluated direct control, pattern recognition, and continuous control modes. With Ariza and Pearce (2022) emphasizing open-source hardware and software platforms [49], accessible, reasonably priced solutions remain a significant focus in prosthesis research. As observed by Gonzalez et al. (2022), where variations in children’s muscle contraction patterns affected prosthesis effectiveness [50], hybrid techniques also meet demands, particular in terms of demography. By means of energy kernel-based techniques, Pancholi and Joshi (2022) enhanced feature extraction to manage force-level variability [51].

### 3.4. Practical Prosthesis Control Assessments, Hybrid Sensing and Rich EMG Data Resources

Furthermore making headway are hybrid control strategies. For robotic hand control, Johansen et al. (2021) [47] integrated tongue-based grasp selection with myoelectric signals, therefore reducing job completion times by over 20%. Likewise, Huang et al. (2023) suggested a cross-modal method using type-2 fuzzy logic to merge sEMG with computer vision data, thus attaining improved grip posture accuracy [52]; assessments in home-use settings mirror the increasing focus on practical performance. Using pattern recognition [53], Simon et al. (2023) compared pattern recognition with direct control in multi-articulating prosthetic hands, therefore displaying broader grip usage and stronger functional outcomes. Variations in muscular contraction patterns among young users underline even more the requirement of age-specific designs [50].

Finally, constant control systems provide fresh avenues for fluid and realistic movement. With the LSTM-based continuous control system developed by Huang et al. (2022), it used joint angular velocity in real time for coordinated multi-DOF movements [52]. All things considered, continuous EMG-based prosthetic control studies show notable advances in signal acquisition, pattern recognition, and accessible design. Investigators are turning prostheses into more functional, dependable, and generally accessible solutions by combining adaptive algorithms, multimodal sensing, and low-cost advancements.

Turning now to data-based studies, recent developments in the use of surface EMG (sEMG) and high-density electromyography (HD-EMG) have considerably contributed to gesture detection, biometrics, and HMI. For example, the GrabMyo dataset gathers multi-day sEMG data from 43 individuals executing 16 hand and wrist movements [54], thus addressing the limits of small-scale investigations. Using HD-EMG, another interesting dataset concentrates on finger movement detection in sync with glove-based joint angle data for 12 different motions [55]. Concurrent with this, the NeuroLife^®^ EMG sleeve was used to estimate continuous joint angles and categorize 37 hand gestures with 97.3% sequential gesture accuracy [5]. Robust and adaptable EMG-driven systems in prosthesis, rehabilitation, and robotics are being made possible by these current datasets and processing methods, which span filtering, dimensionality reduction, and ML-based decoding.

### 3.5. Summary

Summarizing the above reviewed works, we can conclude that there have been rapid gains in EMG-controlled upper-limb prostheses. Classic classifiers (SVM, k-NN, and shallow ANNs) already exceed 90% accuracy with standard filtering, but deep CNNs now dominate for their resilience to noise, electrode shifts, and user variability [9,15,18].

1. Adaptation—incremental, domain-adaptive, and DANN-style learning cut daily recalibration and keep performance stable over long-term use [16,29].
2. Cost-aware hardware and open designs—low-cost 3D-printed sockets, magnetic or optical sensors, and turnkey calibration toolkits widen access without sacrificing accuracy [24,27,31].
3. Hybrid control and rich feedback—multimodal fusion (EMG + SSVEP, vision, and tongue) plus haptic/proprioceptive cues accelerate tasks and reduce fatigue [22,43,47].

Explainable AI, transfer learning, and reinforcement-learning controllers further improve interpretability, cross-user generalization, and multi-DOF dexterity [17,19,46]. Together, these advances chart a path toward affordable, adaptive, and user-friendly prostheses; large-scale longitudinal trials are now the critical next step to confirm safety and everyday reliability [48].

A key strength of any system is robust signal processing to create clean, reliable input. However, the inherent instability of EMG signals due to noise, fatigue, and electrode shifts remains a persistent weakness. Early machine learning models showed high accuracy in labs but failed to generalize to real-world conditions, requiring frequent recalibration.

State-of-the-art deep learning models offer superior accuracy by automatically learning complex features. Their primary weaknesses are their “black box” nature and high demand for data and computational power. To counter these issues, adaptive strategies like incremental learning help models adjust to signal changes over time. Hybrid systems further strengthen performance by fusing EMG with other sensors like computer vision for improved context and accuracy.

The trade-off for these advanced solutions is often increased system complexity and cost. XAI is emerging to interpret model decisions and diagnose problems. Concurrently, a significant research focus is on developing low-cost, user-oriented systems to improve accessibility. While this approach broadens availability, it often involves a necessary sacrifice in performance. Ultimately, the field seeks to balance high-performance control with the development of practical and robust solutions for amputees.

## 4. Closed-Loop Feedback Control of Upper-Limb Prosthesis

Numerous control approaches have been developed to control upper-limb prostheses. Widely used control strategies include classical PD and PID controllers, as well as AI techniques ranging from fuzzy logic to machine-learning methods, including deep learning. In this section, we discuss applications of the aforementioned control solutions in details and provide useful guidance for further directions.

### 4.1. Traditional Methods

In the early stages, prostheses developed for the upper limb were primitive in structure, and were simply controlled by “ON” or “OFF” states depending on EMG signals, the control is achieved by a pair of agonist/antagonist muscles used to directly open and close the hand [56]. However, being open-loop control systems, the functionality of such prosthetic designs was not sufficient to mimic most of the movements of a natural human hand. Usually, the open-loop control system for hand prosthesis cannot guarantee satisfactory performance by resulting in a large overshoot in the output behavior. To mitigate this issue, researchers in [57] proposed a tactile feedback scheme system with proportional and derivative (PD) control to obtain an accurate grasping action of an artificial hand. The PD control was utilized as a finger’s position control in [58,59]. The parameters of the PD controller were tuned to obtain a system response time of less than one second. A digital PID controller was designed to control a direct-current (DC) motor in [60]. This PID controller’s main purpose was to control the position, speed, and current in order to enhance the accuracy of power control and position. In [61], a hybrid method which combines ANN, SVM, and PID control was presented to enhance the extension and flexion movements of an elbow. In this study, the PID controller was chosen as a main feedback controller in order to facilitate the better motion of the joint in the elbow and forearm. A prosthetic solution based on non-invasive functional electrical stimulation (FES) and controlled via hybrid PD-Fuzzy logic control system was presented in [62]. This solution features a real-time proprioceptive feedback system based on tactile sensors. The PD controller is commonly used in the lower-level control of a prosthesis. A reference command generated in a high-level controller is sent to the PD controller to build a closed-loop feedback system. As a feedback signal, one can use the signal from tactile force sensors to compare it with the reference EMG signal value. From this, it is clear that the accuracy of the feedback control system highly depends on the accuracy of two sensors used in the system, especially of an EMG sensor which may have low accuracy due to the fatigue and distraction of muscles. The inclusion of an integral term in a PID control algorithm can negatively impact performance due to wind-up effects, particularly during large or sustained errors. Nevertheless, a PID-like control structure is often preferred for lower-level control tasks, where faster response and stability are prioritized. However, it is important to note that PD control alone cannot eliminate steady-state errors, as it lacks the integral component necessary to correct constant offsets in the output.

### 4.2. AI-Enabled Control Methods

As the number of degrees of freedom (DoFs) increases, controlling the upper-limb prostheses becomes increasingly complex. The challenge intensifies when multiple movements must be executed simultaneously, a scenario where traditional control methods often fall short. A common workaround is to control one DoF at a time, with the ability to switch between DoFs based on predefined thresholds, though this approach limits natural and intuitive movement [63]. The use of EMG sensors in prosthetic control has enabled the realization of multi-degree-of-freedom (multi-DoF) control, allowing for more complex and natural limb movements. AI-based control is commonly employed to infer user intent from EMG signals and to generate target commands for actuator control. However, the overall control system typically functions in an open-loop configuration unless enhanced with specialized sensors such as position, tactile, or force sensors. Achieving closed-loop control in upper-limb prostheses often involves integrating AI-based techniques with conventional control methods, enabling real-time feedback and improved adaptability to user intent.

**Figure 3:**
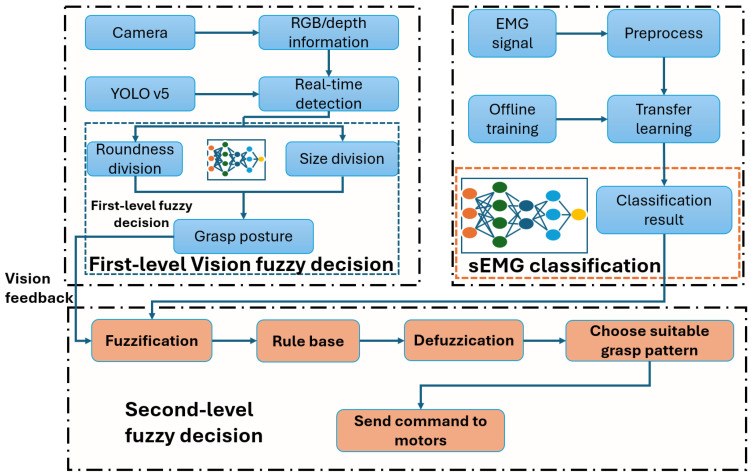
Proposed framework based on the vision-based type-2 fuzzy decision strategy for posture recognition of the grasp [52].

A fuzzy logic control (FLC) is used to propose a new prosthetic control methodology for people with transhumeral amputation in [52]. The proposed framework consists of the prosthetic system, the classifier based on EMG sensors, a decision-making system based on grasp posture, and control scheme based on FLC of type-2. The proposed system was designed to resolve issues with the movement function of a lost elbow and the wrist’s part. The control system comprises a vision-based first-level fuzzy decision strategy, sEMG classification, and a second-level fuzzy decision strategy. The first-level system uses images from a camera on the prosthetic socket, processed by YOLOv5 to classify objects by roundness and size into fuzzy sets. These classifications serve as inputs to the first-level fuzzy system. Simultaneously, real-time sEMG signals are mapped to fuzzy sets and fed into the second-level fuzzy system, which then generates motor commands. Vision aids in low-light and occlusion scenarios, while sEMG compensates for fatigue and distraction. Fuzzy logic replaces fixed PID control, enabling dynamic adaptation to user and environmental changes. A framework of the proposed architecture is shown in Figure 3.

In [64], the control system is structured in two levels: at the higher level, the kNN algorithm classifies hand gestures based on features extracted from EMG signals; at the lower level, the kNN output serves as input to a fuzzy PD controller, which generates the actuator commands. Using the PD and fuzzy logic controllers in a hybrid mode helps to improve the robustness of a control system since the fuzzy logic can mitigate sensor noise, biological variability, and inconsistent EMG signals.

**Figure 4:**
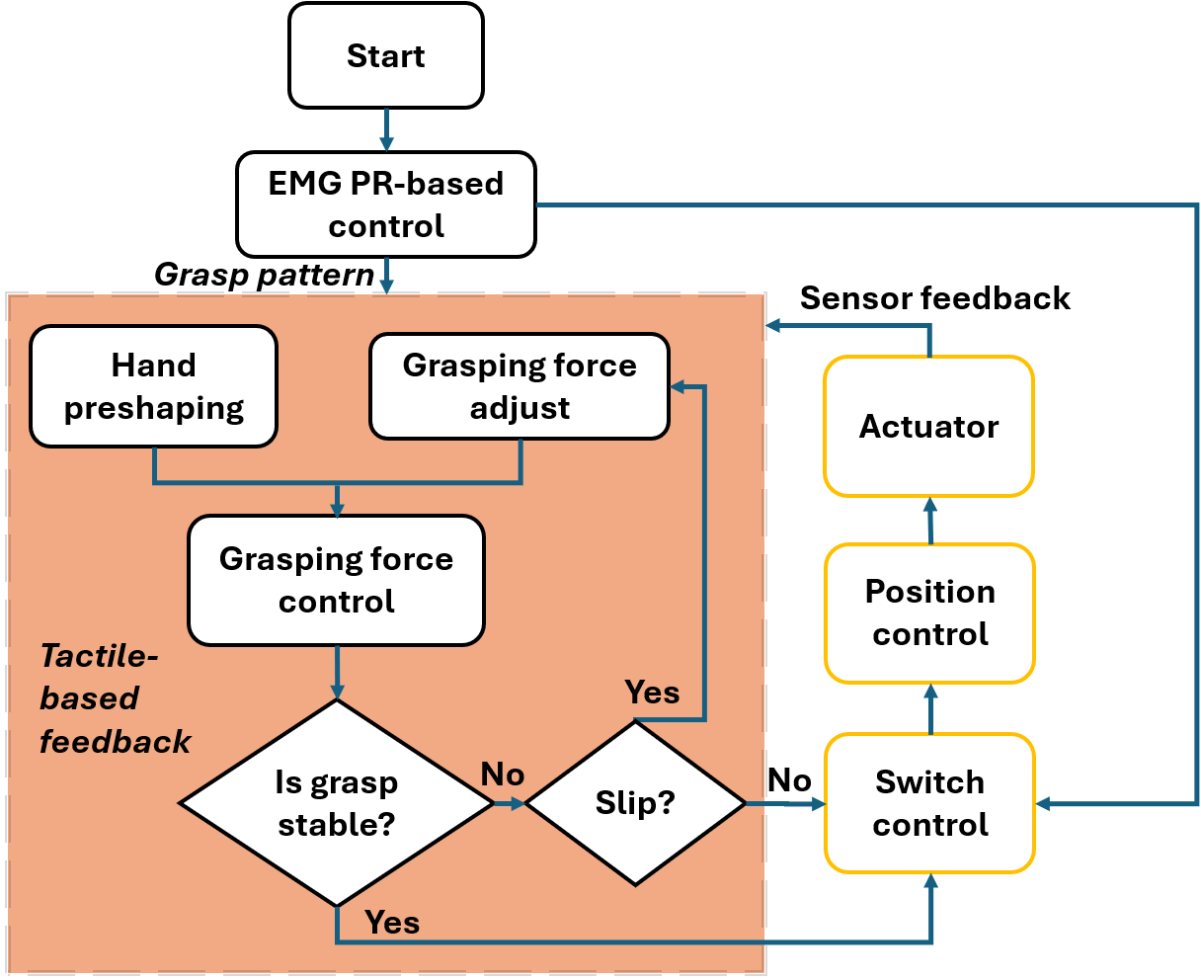
Block scheme representation of the closed-loop feedback control presented in [66].

A cross-modal-based integration control algorithm was proposed in [65] for a prosthetic system for human with humal amputation. To implement the proposed control system, sensor data from the EMG sensor, IMU sensor, and tactile sensor were used. The NN was utilized to train model offline and the pattern recognition was performed online based on the trained model. As a result, the intended instructions were sent to motors based on the obtained motion state. EMG-based PR control approach was introduced in [66] to correctly classify patterns of a grasp action. In this work, the PR control unit receives the data from the EMG sensors and generates the corresponding pattern for a grasp action. The hand preshaping block results in the reference commands for the position and velocity, which are used as inputs for force controllers in the next stage. Thanks to the tactile sensors, the grasping controller regulates the force needed to grasp in real time. In the next step, the slip during the grasping of the object is checked by the grasping force controller. Based on the existence of the slip, more force value is applied in order to resolve a slippage. Finally, the control switch block reenables the PR algorithm when the object is released from the prosthetic hand (Figure 4).

Another example of the AI-based approach can be seen in [67]. This work proposes a so-called “action control” method to a real-time and independent regulation of multiple fingers. In this approach, the EMG signals are translated into a six-dimensional vector with discrete values. The elements of the vector represent one of the existing DOFs, and three different states could be reached by each element, i.e., open state, close state, and stall state. The linear discriminant analysis or LDA is utilized as a classifier to train each existing DOF. The kNN classification method is used as a main classifier in [68]. After training the model, the signals of a selected pose are then used as control signals for the actuator control.

Despite different AI-enabled methods existing in the literature, there are still open research questions relating to the reduced accuracy of EMG sensors due to muscles fatigue and distractions. To overcome such issues, the sensor fusion approach could be used as in [52]; however, it results in a complicated algorithm and bulky design of an upper-limb prosthesis. Furthermore, issue related to power consumption need to be resolved; for instance, ref. [69] reported approximately three hours of autonomous operation of the proposed prosthesis without recharging. EMG-based approaches are most commonly utilized sources of signals in AI-enabled methods; however, EMG sensors cannot guarantee precise outputs when a broad range of limb positions are required by users. For instance, EMG-based control can degrade when a limb’s position becomes different from the position at which the controller was already trained. This, in turn, leads to undesired movements of the wrist and hand [70]. Although there are studies that implemented the fusion of EMG and visual signals from a camera to improve the classification accuracy of the grasp pattern, those approaches are limited to the physiological characteristics of the amputee when obtaining optimal control [71]. A promising direction for enhancing AI-enabled control systems is the integration of inertial measurement units (IMUs). Incorporating IMUs can significantly improve the tracking performance by providing more robust and accurate motion data, leading to more precise and responsive prosthetic control [72].

Visual–tactile sensor fusion is emerging as a promising approach for achieving precise control through AI-enabled feedback mechanisms. This method facilitates adaptive grasping by leveraging safety-critical algorithms such as control barrier functions. Zhijun et al. proposed a safety-critical control framework for vision-based manipulation and grasping, demonstrating that vision feedback enables the system to detect obstacles in cluttered environments and accurately determine object positions and grasping patterns [73]. These encouraging results suggest that the proposed solution could help reduce the cognitive load on amputees and potentially expand the applicability of upper-limb prostheses to individuals with visual impairments.

While numerous control methods have been proposed, their practical implementation is highly dependent on the design and configuration of the transmission mechanism. Accordingly, the following section examines various transmission systems utilized in the development of upper-limb prostheses.

## 5. Transmission Mechanisms

Different studies used various mechanisms to actuate the parts of a prosthetic hand. In each cases, there are different purposes and functionalities of an used mechanism. Some research works aimed to propose flexible, multifunctional, and practical prostheses, which reflect the human demands, while some of the works focused on proposing a prosthesis to be used to accomplish certain tasks which does not require a complicated driving mechanism.

### 5.1. Tendon Based

The most difficult aspects in designing robotic fingers are the configurations that replicate the natural finger and its control, the creation of a robust system and actuation mechanism, along with the incorporation of control strategies and sensing solutions. Generally, the term “actuation” is used to define a motion in the robotic fingers, whereas the term “control” determines a precise and organized movement of the fingers [74]. To reduce the number of actuators and design lightweight prosthetic hands, different approaches to achieve underactuated robotic systems have been studied so far. The underactuated systems are attractive to design flexible grasping, and they are also convenient to develop biomimetic robotic fingers with the functionality of the flexion of the joints responsible for proximal interphalangeal (PIP) as well as distal interpahalangeal (DIP) parts of the finger [75]. Li et al. proposed a tendon-driven robotic finger with variable stiffness in [76]. An adjustable cantilever mechanism is utilized in the actuation system, and this approach creates opportunity to control joint positions and stiffness, which, in turn, enhance the flexibility and safety of the robotic hand during the interaction with objects of different shapes and stiffness. The tendons are required to control both the positions and stiffness of the joints separately. The branching tendon mechanism is used to control three joints in a robotic ginger with only two motors in [77]. Such a design provides more efficient and versatile movement of the finger. To enhance the adaptability and flexibility of the twisted and coiled (TCP) actuators, tendons were integrated in the design of the anthropomorphic hand proposed by Wang et al. [78]. The proposed design could show sufficient performance by achieving 33 different grasping capabilities and passing the Kapandji test. The dual-mode actuation system presented in [79] works in both fluidic and tendon-based actuation modes by twisting a flexible tube filled with gas, liquid, or mix of both. The proposed design could achieve significant results in the bending angle and strong blocking force of the soft robotic finger. Compared to traditional fluidic elastomer actuation schemes (FEA), the bending angle could be increased up to 150% and the blocking force by up to 134%. The tendon-based actuation was used to design the bio-inspired robotic finger by Zhu et al. [80]. The design incorporates a ligamentous joint that ensures anisotropic variable stiffness, showing better finger adaptability, dexterity, and stability. Chang et al. replicated the effective swing mechanics of a human hand in the proposed prosthetic hand, which makes it applicable for sport games, such as golf, in which swinging motions are required [81]. The actuation scheme of the prosthetic hand uses a differential actuation system to decrease the complexity of the actuation. The differential system in the prosthetic hand includes mobile pulleys, stationary pulleys, and rods for guiding sliders. The prosthetic hand can increase the swing speed at 90 rpm (increase by 19%), which helps to enhance performance in sports. A double-acting soft actuator (DASA) was used to design a portable and high-compliant soft robotic finger in [82]. The actuation system uses a mechanism of the compressing bellow that contracts and pumps out fluid, which provides both fluidic power and tendon-driven force concurrently. A tendon-driven mechanism was utilized in the work of Estay et al. [83] to reduce the weight of the prosthesis and minimize the amount of signals to manipulate it. In overall, the robotic hand mechanism consists of three actuators: one actuator controls the little, annular, and middle fingers, the next actuator controls the index finger, and the last actuator controls the thumb finger. The fingers of the proposed design can adapt to the various shapes of objects by making it a flexible and secure grip. The anthropomorphic hand with an antagonistic actuation mechanism was designed by Min and Yi, who proposed parallel cables for the joints of fingers driven by a single motor. Moreover, the PIP and DIP joints are underactuated via using a flexible tension spring. The antagonistic actuation is conveniently achieved by utilizing a parallel knuckle mechanism which provides two DOFs for a joint [84]. To address the problem with extending a task space for a gripper and improving its grasping performance, Yoon et al. proposed tendon-driven underactuated robotic fingers in their work [85].

The proposed design is represented by revolute–prismatic (RP) modules with a coil spring serving as the phalanges. In this design, the revolute joints are responsible for the flexion and extension movements of the finger parts, while the prismatic joints are used to achieve elongation and contraction of the finger length. Each part of the module has an elongation part due to the use of the prismatic joint, and flexion part due to the application of the revolute joint. Two tendon cables serve as the antagonistic pairs and provide control of both angles of the joints and lengths of the links. The experimental results show that the elongation of fingers can improve the maneuverability and dexterity of the prosthetic hand to accomplish a various range of tasks.

### 5.2. Linkage-Based

Kashef et al. presented a comprehensive review of 28 linkage-based finger transmission mechanisms based on articles published between 2000 and 2019 in [12]. Based on the provided information, it is stated that the widely used transmission mechanism to connect the prosthetic fingers is a tendon-based or linkage-based type. Despite the former type being attractive in terms of its light weight and simplicity in structure, the latter type is preferable when one needs to design a prosthetic finger that can be durable for high forces and capable of operating with higher accuracy [12].

In general, several important criteria should be addressed to evaluate the mechanism used in the prosthetic fingers, and Kashef et al. discussed eleven important criteria to analyze the different proposed mechanisms in terms of grasping and physical point of view. The criteria that determine grasping characteristics are named natural motion, shape adaptivity, pinching motion, stability, force isotropy, workspace, and stiffness, whereas the weight, number of phalanges, compactness, and manufacturing process are considered to define the physical properties. Suthar et al. discussed the different mechanical designs of prosthetic fingers and provided a detailed overview of the state of the art in prosthetic fingers by highlighting the most important trends, challenges, and current research gaps [74]. Based on the material used in the robotic finger design, a mechanism type can be categorized as rigid, semi-rigid, and soft mechanism, while in terms of actuation type, the actuators can be of the rigid, semi-rigid, or soft type [74]. Different rigid mechanisms have been proposed in many research works. Li et al. [86] proposed a linkage-based mechanism for a robotic hand with three fingers. The robotic hand utilizes a spherical four-bar linkage mechanism to transmit power to fingers which, in turn, include a planar linkage responsible for grasping action. Furthermore, two actuators are set in parallel and used to achieve flexion/extension and abduction/adduction motions. Based on the different analysis and tested scenarios, the proposed prosthetic hand showed 92.5% of accuracy for grasping performance. A single actuator-based robotic hand with five fingers was presented in [87]. It is stated that multiple actuators used in a prosthetic hand design usually lead to a decrease in the grip force, and most of the commercial prosthetic hands utilizing multiple actuators show a pinch force ≈ 11–30 N which is less than the recommended value of 68 N. The prosthetic hand in [87] uses a single actuator which is connected with four fingers and a thumb via two sets of four-bar linkage mechanisms. The proposed single-actuator-based hand includes 38 revolute joints, in which 6 revolute joints are used in the finger part and 8 revolute joints are placed in the hand part. Furthermore, two prismatic joints are chosen to move the fingers and the thumb. To provide a tripod function and achieve precise grip functionality, the researchers placed the thumb between the index finger and middle finger. Although the proposed design showed results close to the required specifications, it requires further improvement to provide more practicality in terms of weight, control, and size. A robotic finger based on the multiple linkage underactuated mechanism was proposed in [88]. Hailiang et al. focused on achieving accurate pinching and strong grasping functionalities of the robotic finger. The proposed design has a three-fingered structure with three working resumes as centering grasping, parallel grasping, and two-finger-based symmetrical pinching. The results of the tests conducted to check the contact force, grasping functionality, and load-lifting capacity showed that the linkage-based mechanism could guarantee uniform force distribution, good precision in the grasping of small and weak objects, and strong load-lifting capacity.

### 5.3. Pneumatic

Recently, the pneumatic type of actuation has been used widely in robotic applications. This type of actuator is becoming popular due to several characteristics such as flexibility, light weight, and safety related to human interactions [89]. According to the surveyed literature, pneumatic actuators are commonly used in soft robotics applications [90]. To resolve the limitations existing in most pneumatic actuators related to limited mode of operation (bending or twisting), Chen et al. [91] proposed a new wave-shaped soft actuation mechanism. The wave-shaped actuators allow for improved compliance and adaptability. The proposed system uses the compressed air to pump up the actuators. Due to the increase in the air pressure, the wave-shaped actuators expand and bend, and as a result, a grip motion is created. The bending motion of the actuator is stable and reliable, which provides better grasping results of objects with different shapes and sizes. Different scenarios for grasping objects were tested. Based on the tests, the proposed four-finger robotic hand solution was able to successfully grasp objects like oranges, bottles, drink cans, tissue boxes, and hollow jars. Dual-module soft robotic actuator (SPA) with variable chamber height (DMVCHA) was presented in [92]. With the aim to enhance grasping and its reliability, the proposed scheme works in two grasping modes, i.e., pinching and enveloping. Depending on the type of grasping mode, the actuation mechanism can work as a pneumatic network actuator with a variable height of the chamber by providing a large force on the output, or be a soft gripper with high passive flexibility by allowing to grasp objects with a small and average size. Positive–negative pneumatic actuation is used to design an anthropomorphic hand with multi-degree-of-freedom motion in [93]. The positive pressure is supplied to inflate the soft bellows, which results in bending of the fingers, whereas the negative air pressure (vacuum) is used to deflate the bellows in order to extend the fingers. Thus, this combination of the positive and negative actuation helps to mimic the natural flexion and extension motions of human fingers. The positive–negative actuation facilitates the prosthetic hand to perform dorsflexion motion and is considered an expansion of its range of motion.

An anthropomorphic hand with 21 degrees of freedom is proposed in the work of Mei et al. [94]. The pneumatic actuation in the proposed work is based on the open-loop scheme in order to regulate the air pressure, i.e., the controller regulates the PWM signal to the proportional pressure valve based on the command received from the PC.

Pneumatic actuation could be applicable for robots with a compact size due to its flexibility, high-power density, and economic cost [95]. However, using a traditional air compressor in a pneumatic actuation might be considered a main obstacle to apply such an actuation type in compact robotic devices. To tackle this issue, Kim et al. proposed a method based on the decomposition of hydrogen peroxide [95]. They designed a dual-mode actuation approach in using pneumatic actuation to complete 1200 grasps, the number of grasps performed by people in daily activities. The designed robotic finger showed a flexion speed of 468 deg/s and a fingertip force of about 29.1 N.

Severity in the controlling slippage between the finger’s surface and grasped object is typical for soft robotic hands. To resolve this difficulty, Nishimura et al. proposed a 1-degree-of-freedom (DOF) actuation scheme for soft robotic hands [96]. The proposed scheme was developed such that it can control both the injection of the lubricant and finger motions via the 1-DOF actuation scheme. The switching strategy is achieved by controlling the airflow used for actuation. A low airflow level is supplied to provide finger motion, and the injection is achieved via high level airflow. The performance of the robotic hand is verified via placing, manipulation, and grasping tests.

It is noticed that the palm in the robotic hand plays a crucial role in reinforcing the grasping capability and tactile sensation. Thus, full contact between the palm and grasped object can improve grasping and help the robot hand to accurately adapt to objects shapes. Pneumatic actuation is used in the design of the soft robotic hand, which consists of four soft robotic fingers and an inflatable palm [97]. Different tests have been conducted for validation of grasping flexibility and perception. Furthermore, a control algorithm based on tactile information is proposed to guarantee stable grasping motion. Table 2 summarizes the details of each actuation mechanism presented during the surveying period.

Several recent studies have introduced transmission mechanisms based on tendon-driven, linkage-based, and pneumatic actuation as discussed in this section. While both tendon-driven and linkage-based mechanisms are widely employed in the control of robotic hands, the majority of proposed solutions favor tendon-driven systems due to their flexibility and efficiency. Table 2 shows that most of the tendon-driven systems are actuated via electric motor, which could be beneficial in terms of precise control and easiness of control compared to other sources of actuation. However, it is known that any electric motor leads to an increase in overall weight. Therefore, there is still a trade-off between the number of electric motors and overall weight in the design of prosthetic hands. Despite such trade-offs, the tendon-driven mechanism can provide flexible and efficient driving solutions for future prosthetic development. Flexibility improvement could be seen in [76,77,78,80,82], where the adaptability and dexterity of a robotic hand were improved by joint stiffness control, improved bending angle, and blocking force. This, in turn, will positively impact prosthetic development via continuous stiffness regulation, improved fingertip feasible force space, and the constrained control of two joints in a finger. Reducing the number of actuators (electric motors) in a tendon-driven robotic hand creates additional space for integrating hardware components essential to developing AI-based algorithms, enabling the design of lightweight, cost-effective, fully functional, and intelligent prosthetic hands. Notably, the lightweight design of tendon-driven mechanisms makes them particularly advantageous in systems employing multi-modal sensor fusion, where minimizing mechanical complexity is critical. This synergy enhances the quality of feedback signals, which is vital for the performance and reliability of AI-driven control strategies. Although linkage-based mechanisms show promise, their overall complexity and weight pose significant limitations for widespread application in prosthetic hand development.

Pneumatic actuation, on the other hand, is predominantly used in soft robotics. However, with ongoing technological advancements, its application is expected to expand significantly, including in the control of rigid-body robots. The following section will explore various tactile sensing technologies, which are crucial for developing fully controllable and practical robotic hands.

## 6. Sensor Technologies for Upper-Limb Prostheses

Recent developments in actuator design have allowed a wide range of finger and hand structures that closely resemble the movement characteristics of a natural limb in upper-limb prostheses. However, actuation alone is inadequate for fully realistic prosthetic control; closed-loop systems require comprehensive sensory feedback to regulate grip dynamics and adapt to task variations. As a result, sensors are critical in upper-limb prostheses, providing the real-time contact and kinematic data required for intuitive and reliable performance. A detailed understanding of the sensing principles is essential for the optimal selection, placement, and integration of these devices. Sensors used in upper-limb prostheses typically fall into two categories: tactile sensors and proprioceptive/environmental sensors. Tactile sensors are designed to detect cutaneous sensations, including normal and shear forces, vibrations, and temperature changes. In contrast, proprioceptive and environmental sensors are devices such as joint angle encoders, inertial measurement units (IMUs), probes embedded within sockets, and structural accelerometers, which measure joint movements, orientation, structural forces, and environmental interactions. Although these sensors can be used to provide sensory feedback to the individual, this typically requires additional mechanisms such as FES, nerve interfaces, or haptic devices. However, this chapter focuses specifically on the sensor role within the closed-loop control architecture.

### 6.1. Tactile Sensors

Tactile sensors are essential components of modern robotics, allowing devices to perceive and respond to physical stimuli such as pressure, texture, and temperature. Inspired by the human sense of touch, these sensors provide a significant function in a variety of applications, particularly in prosthetics, where they improve the ability to perform delicate tasks, such as grasping objects or detecting slippage in real time [101]. Recent advances in tactile sensing technologies have led to a wide range of sensor types, each based on different principles and designed for specific applications. Table 3 provides an overview of the main types of tactile sensors, highlighting their materials, unique features, and practical applications. To provide a clearer comparison, Table 4 outlines the key advantages and limitations of these technologies. The use of tactile sensors is particularly important in environments that pose durability challenges, such as exposure to abrasion, moisture, and extreme weather conditions [102,103,104]. Harsh climates and environmental factors, including electromagnetic interference and static electricity in dry conditions, can affect the performance and reliability of these sensors [105,106]. Therefore, selecting tactile sensors made of durable materials and designed to withstand these conditions is essential to ensure reliable performance, particularly in Kazakhstan’s diverse and often extreme climate.

#### 6.1.1. Capacitive Tactile Sensors

Tactile sensors are essential for detecting pressure and shear forces in prosthetics, enhancing functionality and user experience. Capacitive tactile sensors are one of the most common types, which detect changes in capacitance caused by mechanical forces, altering the configuration of two conductive plates separated by a dielectric material. Changes in capacitance are translated into electrical signals, allowing for precise touch or force detection. The sensitivity and functionality of capacitive sensors depend on the design of the dielectric layer design, the electrode materials, and the structural configurations [107]. Advancements in capacitive tactile sensor technology focus on improving sensitivity, durability, and scalability while addressing material performance, environmental stability, and high pressure reliability challenges. Ionic hydrogel electrodes offer great stretchability and long-lasting strains, making them optimal for soft robotics. However, they have lower luminous efficiency than commercial devices, limiting some interactive applications [107]. Materials such as carbon-doped polydimethylsiloxane (PDMS) and cellulose nanocrystals (CNCs) modified with tannic acid have been studied for dual-mode detection and self-healing properties. However, carbon-doped PDMS-based sensors face challenges in reliably detecting nonconductive objects and can be prone to misinterpretations caused by side sensitivity and the unintended activation of proximity sensors [108]. CNC-based sensors feature ultrastretchability and antibacterial properties, making them suitable for wearable applications, but large-scale manufacturing remains a challenge [109]. Integrating multi-wall carbon nanotubes (CNTs) and silver nanowires (AgNWs) into flexible structures improves sensitivity over a wide pressure range. Multi-wall CNT-based sensors are designed to ensure durability and sensitivity at low pressures [110]. However, their performance decreases under high pressure. AgNW sensors utilize crack-enhanced designs that improve sensitivity, especially in wearable applications, although they are prone to degradation due to liquid evaporation [111]. Capacitive sensors made of graphene oxide and photoreduced graphene oxide have shown rapid responses and high sensitivity for moisture detection. These sensors work in a noncontact mode, making them suitable for detecting environmental stimuli, although their performance may be affected by high temperatures [112]. Furthermore, poly(vinylidene fluoride-cotrifluoroethylene) sensors with interlocked nanocone arrays provide a low-cost option for pressure sensing, although their sensitivity decreases under higher pressures [113].

PDMSs and CNTs are widely used materials in the design of capacitive tactile sensors to maintain functionality under diverse environmental conditions. PDMS is particularly valued for its excellent thermal stability and flexibility, which maintains its mechanical properties even at very low temperatures because of its low glass transition temperature. This flexibility minimizes the risk of mechanical failure in cold environments. In addition, PDMS has low surface energy and hydrophobic characteristics, which prevent dust accumulation and improve performance in applications requiring cleanliness. However, its insulating properties can cause static charge buildup, which can be addressed by using conductive fillers or applying surface treatments that improve antistatic performance. Similarly, CNTs have high mechanical strength and thermal durability, making them useful for sensors exposed to subzero temperatures. Combining them into polymers can improve sensor durability and flexibility while also using their high electrical conductivity to reduce electromagnetic interference.

#### 6.1.2. Piezoresistive Tactile Sensors

Piezoresistive tactile sensors detect mechanical pressure by translating resistance changes into electrical signals, making them highly suitable for applications in wearable devices and robotics. These sensors operate on two primary mechanisms: geometric changes and resistivity changes within conductive materials such as CNTs, graphene, or metal nanoparticles. Pressure alters the internal structure of the material, affecting electrical conductivity, which enables precise force detection in various ranges [114]. Hybrid materials such as MXene/CNT composites and liquid metals embedded in microchannels demonstrated multitouch sensitivity and mechanical flexibility, making them effective in electronic skin and soft robotics [115,116]. Introducing materials such as molybdenum disulfide in active matrix designs improves the accuracy of pressure detection by reducing interference between sensors [117]. Similarly, liquid-metal-based sensors combined with PDMS matrices provide high sensitivity to bending and stretching forces [118]. Advanced designs using micropyramid structures or hollow-sphere microstructures further improve sensor performance by optimizing the redistribution of stress across the sensor surface, resulting in high sensitivity and low hysteresis [119]. Furthermore, Velostat-based sensors provide reliable three-dimensional force detection, which makes them useful for smart gloves and robotic grippers [114].

The integration of advanced materials is critical to improving the performance and reliability of piezoresistive sensors. CNTs provide mechanical strength, thermal durability, and electrical conductivity and are particularly effective at enhancing sensor functionality at subzero temperatures. When combined with polymers or metals, CNTs improve flexibility and toughness, ensuring consistent performance even in extreme cold conditions. Similarly, nanocarbon composites are utilized to address the problem of reduced electrical conductivity induced by material shrinkage at cold temperatures. These materials also provide resistance to environmental pollutants, such as dust and dirt, which can interfere with signals and cause mechanical wear.

#### 6.1.3. Piezoelectric Tactile Sensors

Piezoelectric tactile sensors take advantage of the inherent capacity of specific materials to create electrical charges when subjected to mechanical stress, known as the piezoelectric effect. Materials such as lead zirconate titanate (PZT), polyvinylidene fluoride (PVDF), and barium titanate are widely employed in these sensors to detect dynamic forces, including vibrations and pressure changes. An important advancement involves the integration of PZT with a soft silicone substrate, which results in an ultrathin, stretchable sensor with a 100-fold increase in sensitivity over older designs. These sensors have significant advantages, including conformability to skin surfaces, stretchability, rapid response times, and low hysteresis, providing constant performance over time [120]. However, fabrication procedures pose a challenge since the sensors can crack during the transfer printing stage and their accuracy is reduced when applied to extremely curved surfaces. Another major breakthrough is the combination of PDMS and PVDF to achieve crosstalk-free sensing. This multilayer design utilizes a unique row-and-column electrode layout to prevent signal interference across sensor channels. PDMS-PVDF sensors have high sensitivity and maintain performance stability over many cycles at a normal force of 15N [121]. Despite these benefits, fabrication problems such as bubble formation during spin-coating are present. Self-powered piezoelectric sensors using ultrathin polyethylene terephthalate generate electrical signals from mechanical forces. However, their limited sensitivity to static forces restricts their use in static pressure applications [122]. Recent advances in nanofiber-based piezoelectric sensors have used electrospinning techniques to improve performance. PVDF nanofibers containing barium titanate (BTO) particles and CNT have a stronger piezoelectric output due to an increased concentration in the β phase. These nanofiber-based sensors detect pressures and can withstand high loading cycles without degradation [123]. The integration of PZT nanofibers into PDMS composite films has enabled sensors to take advantage of piezoelectric and triboelectric effects, enhancing sensitivity and measurement range. Their micro-frustum array design increases the surface area for charge generation, resulting in high sensitivity and enhanced pressure measurement capabilities [124].

Despite advances in piezoelectric tactile sensors, various challenges limit their widespread practical adoption. Although PZT materials maintain their piezoelectric properties in cold environments and offer resistance to dust infiltration, they remain vulnerable to static electricity, especially in dry, cold conditions. Similarly, PVDF exhibits flexibility and operational stability at low temperatures, making it more suitable for outdoor applications. PVDF has low surface energy and hydrophobicity which reduce dust adhesion and moisture accumulation, which is advantageous for wearable sensors. However, both materials are prone to electrostatic discharge, which can cause signal interference or damage sensitive components.

**Table 3:** Examples of tactile sensor technologies with distinct principles.

Sensor Type	Materials	Tactile Applications	Unique Features	References
Capacitive Tactile Sensor	Graphene oxide and photoreduced graphene oxide	Moisture detection	All-graphene device enabling noncontact moisture sensing via femtosecond laser direct writing for single-step, eco-friendly fabrication	[112]
	Cellulose nanocrystals modified with tannic acid and silver nanoparticles	Strain detection	Ultra-stretchability exceeding 4000% combined with rapid self-healing and antibacterial properties	[109]
	Poly(vinylidene fluoride-co-trifluoroethylene)	Pressure detection	Interlocked asymmetric-nanocone arrays (using unpolarized P(VDF-TrFE)) that localize stress at apexes for superior and scalable sensing performance	[113]
	Carbon Nanotubes combined with Polydimethylsiloxane	Pressure detection	Gradient micro-dome architecture achieving simultaneously high sensitivity and ultrawide linearity, enabling non-overlapping capacitance signals	[125]
	Multi-wall Carbon Nanotubes combined with Polydimethylsiloxane	Pressure detection	Hierarchical bionic spine–pillar architecture offering heightened low-pressure sensitivity and a broad high-pressure range.	[110]
	Polydimethylsiloxane	Pressure detection	Biomimetic gray kangaroo leg microstructure, leveraging rapid bending–releasing mechanisms for ultra-high sensitivity and wide pressure range	[126]
Piezoresistive Tactile Sensor	Galinstan (liquid metal) embedded in microchannels within a polydimethylsiloxane matrix	Pressure detection	Embedded Wheatstone bridge achieving high sensitivity (sub-50 Pa resolution) and simultaneous temperature self-compensation	[115]
	Piezoresistors combined polymeric packaging	Slippage detection, Force sensing	Slippage detection using raw voltage alone, enabling rapid parallel force and slip estimation	[127]
	Molybdenum Disulfide	Pressure detection	Active-matrix design with integrated TFTs reducing crosstalk among sensing units while maintaining a wide, linear pressure range	[117]
	MXene/Single-Walled Carbon Nanotubes/Polyvinylpyrrolidone conductive film	Pressure detection, Voice recognition	Dendritic-lamellar architecture delivering high void space for extreme low-pressure detection (0.69 Pa) and robust structural integrity	[116]
	Conductive silver threads (resistive sensing), Flexible fluidic tubes (pressure sensing)	Pressure detection, Temperature Sensing	Dual-modality sensing (resistive + fluidic) with decoupled signals for pose and pressure while keeping electronics off-hand	[128]
	Carbon Nanotubes combined with Polydimethylsiloxane	Strain detection in underwater	Biomimetic swim bladder design integrating sensing and pneumatic actuation for underwater applications with stable morphability	[129]
	Nanocarbon-polymer composite	Pressure detection	Cross-striped nanocarbon-polymer composite enabling ultra-fast and high-spatial-resolution tactile sensing via screen printing	[130]
	Velostat (polyethylene-carbon composite material)	Detecting normal and shear forces	Sandwich-structured Velostat sensor supporting both large normal (0–12 N) and shear (0–2.6 N) sensing, incorporated in a glove for real-time wireless feedback	[114]
Piezoelectric Tactile Sensor	Lead zirconate titanate	Pressure detection	Integration of PZT on a soft silicone substrate, yielding around 100-fold sensitivity boost and ultra-fast response, while maintaining high stretchability	[120]
	Lead zirconate titanate with ultrathin polyethylene terephthalate	Pressure detection	Self-powered, ultrathin, and wireless design that directly converts mechanical forces into electrical signals without external power sources	[122]
	Polyvinylidene fluoride nanofibers	Pressure detection, temperature sensing	Single-electrode configuration that maintains a steady-state pressure signal and is area-independent, simplifying microminiaturization and autonomous applications	[131]
	Piezoelectric film (Au/polyvinylidene fluoride)	Pressure detection, Surface texture identification	Self-powered sensor mimicking fast- and slow-adapting mechanoreceptors, capturing complex tactile stimuli across a broad frequency range without external energy input	[132]
	Lead-Zirconate-Titanate Nanofibers with Polydimethylsiloxane composite film	Pressure detection	Dual piezoelectric–triboelectric mechanism with micro-frustum arrays that expands the detection range and enhances sensitivity on highly skin-conformal substrates	[124]
	Polydimethylsiloxane with Polyvinylidene Fluoride	Pressure detection, Slippage detection	Crosstalk-free multilayer row + column electrode design drastically reducing wiring complexity and enabling simultaneous multi-mode stimulus detection	[121]
	Polyvinylidene fluoride nanofiber with barium titanate particles	Pressure detection	Self-powered electrospun fibers doped with CNT/BTO for high β-phase content and a broad detection range, maintaining durability over 12,000 cycles	[123]
	Polyvinylidene fluoride	Pressure detection, Slip detection	Rigid-in-soft truncated-pyramid design, delivering a 1.7 times sensitivity increase without sacrificing flexibility	[133]
Triboelectric Tactile Sensor	Nickel-Fabric Conductive Textile with Polytetrafluoroethylene Film	Pressure detection, Slip detection	Specially distributed electrodes and a gear-based strip to self-power, detect both contact position/area, and continuously track elongation while minimizing environmental noise	[134]
	Polyvinylidene Fluoride	Pressure detection	Concentric dual-mode sensing (triboelectric + inductive) with a shield ring and CNN-based signal fusion, yielding high recognition accuracy	[135]
	Polydimethylsiloxane with Silver Nanowires	Pressure detection	Simultaneous tactile and touchless detection via triboelectric–liquid metal mechanisms, enabling real-time mode distinction and material identification	[136]
	Polydimethylsiloxane with Polycaprolactone Nanofiber Membranes	Pressure detection	Self-powered, biodegradable PDMS/PCL nanofiber design offering biocompatibility, scalability, and consistent triboelectric output under varying environmental conditions	[137]
	poly(vinylidene fluoride-co-hexafluoropropylene), polyvinyl chloride, and titanium dioxide composite film	Pressure detection	Leverages a hydrophobic composite film patterned by sandpaper that provides superior moisture resistance, tripling output performance over bare PVDF–HFP TENGs while retaining flexibility and durability	[138]
	Organic single crystals (Schiff base 1)	Pressure detection	Flexible organic Schiff base single crystals with reversible ion functionalization enabling noncontact operation, high power density, and exceptional endurance over 10,000 cycles	[139]
Electro-chemical Tactile Sensor	Ionic liquid confined in silica microstructures embedded in thermoplastic polyurethane	Pressure detection	Ultra-low-voltage (1 mV) sensor leveraging a double hydrogen-bond network for reversible ion pumping, yielding extraordinarily high sensitivity and minimal initial capacitance	[140]
	Ionic Gel (Poly(vinyl alcohol)) combined with an ionic liquid	Temperature sensing	Completely water-dissolvable, biodegradable temperature sensor featuring optical transparency and mechanical flexibility, facilitating eco-friendly disposal and potential integration with wearable displays	[141]
	Ferroelectric barium titanate nanoparticles encircled with ionic liquid	Tactile memory retention, Pressure detection	Combined pressure sensing and long-term memory in a single device via ferroelectric-assisted ion dynamics (FAID), enabling low-energy (20.9 pJ) operation without separate sensing and memory modules	[142]
Magnetic Tactile Sensor	Titanium Grade 5	Pressure detection	Contactless magnetic force–torque sensing integrated directly into a standard male pyramid adapter with minimal added mass, preserving off-the-shelf prosthetic compatibility	[143]
	Magnetic microparticles (MQP-15-7; Magnequench) embedded in elastomer	Detecting normal and shear forces	Replaceable skin layer decoupled from the magnetic sensing electronics, combined with self-supervised machine learning for high-resolution, adaptable, and low-cost tactile sensing	[144]
	MXene composite with Fe_3_O_4_ nanoparticles	Proximity sensing, Pressure detection	Dual-mode textile sensor with MXene nanosheets distinguishing proximity from pressure via a clear resistance switching point, all fabricated through a simple coating process	[145]
	E-fiberglass/epoxy composite with magnet	Force measurement, Bending moment detection	Two-stage magnetic transduction measuring millimeter-scale deflections on curved surfaces for robust, cost-effective load sensing without the need for high-precision machining	[146]
	Centripetally magnetized flexible magnetic material with NdFeB microparticles	Detecting normal and shear forces	Split-type wireless 3D force decoupling, inspired by biological layering with interchangeable buffer layers to adjust sensitivity and measurement range	[147]
Optical Tactile Sensor	Quadrant photodiodes combined with silicone	Force measurement, slip and friction detection	Pinhole camera–based silicone pillars and a quadrant photodiode providing true 3D force/displacement measurement and high-frequency vibration sensing (up to 1000 Hz), each pillar operating independently	[148]
	Organic semiconductors (rubrene/fullerene diodes)	Pressure detection, position sensing	Reversible rubrene/fullerene diodes functioning as both OLEDs and OPDs, enabling simultaneous light emission and detection without performance degradation or hysteresis	[149]
	Polydimethylsiloxane combined with elastic resin	Pressure detection, Slip detection	Finger-skin-inspired multilayer with optical microfiber ridges, allowing concurrent force and slip detection via wavelet analysis, all fabricated without photolithography or vacuum processes	[150]
	Multimode fiber embedded in a silicone pad	Spatial position detection, force sensing	Single-fiber multimode interference fused with deep learning, yielding high-resolution tactile classification, easily scalable soft silicone design	[151]
	Zinc sulfide–calcium zinc oxysulfide mechanoluminescent hybrid	Pressure detection, temperature sensing	Interference-free bimodal sensing avoiding crosstalk or complex signal processing, with real-time visual emission for pressure	[152]
Vision-based tactile sensor	Camera	Detecting normal and shear forces	Single-layer depth from defocus technique enabling 3D force reconstruction without multi-layer markers, significantly reducing sensor complexity	[153]
	Camera	Pressure detection	Compact, cost-effective, and open-source optical tactile sensor (DIGIT) featuring modular elastomers, streamlined manufacturing, and custom electronics for large-scale production	[154]
	Event-Based Camera	Texture classification	Biomimetic neuromorphic design mimicking human glabrous skin, producing spike-based output via an event-based camera to emulate fast-adapting afferents	[155]
	Camera	Pressure detection	Flexible marker-based vision sensing with tunable PDMS touchpoints, combining high-resolution tactile–visual information in a single, adaptable design	[156]
	Event-Based Camera	Vibration Sensing, Shear Force and Slip Detection	Event-based camera delivering 1000 Hz sampling at 640 × 480 resolution with sparse data, lowering data rates while maintaining high-speed optical tactile sensing	[157]

Moreover, durability under high mechanical strain is a limitation for many piezoelectric sensors, as repeated exposure to bending and stretching can cause fractures, compromising longevity. The integration of BTO particles into nanofiber-based sensors can enhance performance but can weaken the mechanical structure, resulting in loss of continuity.

#### 6.1.4. Triboelectric Tactile Sensors

With significant advantages over conventional sensors that depend on external power sources, triboelectric tactile sensors have become an innovative technology in the field of self-powered sensors. These sensors do not require external power sources since they depend on the triboelectric effect, or contact electrification between materials, to convert mechanical energy into electrical signals. Li et al. presented a hybrid sensor composed of PVDF that incorporates triboelectric and inductive sensing methods. The dual mode sensing capacity allows for simultaneous pressure detection and electromagnetic sensing, and machine learning integration increases object recognition accuracy [135]. However, concerns such as signal saturation and data processing complexity remain. Sensors that use nickel-fabric conductive textiles and PTFE films can detect pressure, slip, and contact position [134]. These sensors are highly compatible with soft robotics because they match their mechanical characteristics with silicone rubber and use ML methods such as support vector machines to improve recognition accuracy. Similarly, a stretchable sensor developed from PDMS and silver nanowires offers both tactile and touchless sensing by distinguishing between contact and proximity stimuli in real time [136]. Furthermore, PDMS-based triboelectric sensors that combine polycaprolactone nanofiber membranes are biocompatible and biodegradable [137]. Flexible triboelectric sensors constructed from PVDF-HFP, PVC, and titanium dioxide composite films have increased hydrophobicity and high electrical output, as well as being able to tolerate humid environments and long-term use [138]. The use of organic single crystals in triboelectric sensors enables noncontact sensing, which reduces mechanical damage and increases sensor lifespan. These sensors have a high power density and long-term endurance [139].

The materials used in triboelectric tactile sensors offer significant advantages but also present challenges that can impact their performance and reliability, particularly in harsh environments. For instance, PDMS demonstrates excellent thermal stability and flexibility at low temperatures as a result of its low glass transition temperature. However, its susceptibility to static charge accumulation can disrupt sensor outputs, often necessitating modifications such as conductive fillers or surface treatments, which increase material complexity. Similarly, PVDF offers remarkable flexibility and robust electrical properties even in extreme cold, making it ideal for harsh climates, yet its dielectric nature may not fully mitigate the risks associated with electrostatic interference. Although these materials have low surface energy and hydrophobic characteristics, which reduce dust and contaminant adhesion, they fall short of solving concerns like material fatigue at cold temperatures or inconsistent signal outputs in low-humidity environments. Despite these limitations, advances such as PVDF-based dual-mode sensors and PDMS-silver nanowire composites show promise in overcoming some of these challenges by including functions such as contactless sensing and increased environmental robustness.

#### 6.1.5. Electro-Chemical Tactile Sensors

Electro-chemical tactile sensors differ from other sensors by their integration of ion dynamics or ionic interactions to achieve ultra-high sensitivity, low power consumption, and multifunctionality, such as combining pressure sensing with features like biodegradability or memory retention. Chun et al. proposed a self-powered ionic sensor capable of generating slow- and fast-adapting signals to detect mechanical stress and vital signs, based on biological ion channels [132]. Bioinspired designs mimic mechanoreceptors developed by embedding silica microstructures in flexible matrices, resulting in excellent pressure sensitivity with low energy consumption [140]. Furthermore, ionic gels have been used to develop temperature sensors with quick response times and features such as self-healing and biodegradability [141]. The integration of sensory functions with memory retention developed a pressure-sensitive sensor that maintains tactile information without continuous power input [142]. Beyond tactile sensing, visual feedback integration has been accomplished with electroluminescent materials that change color in response to inputs.

Electro-chemical tactile sensors face challenges in material stability and durability, with factors such as temperature, humidity, and contamination affecting performance. For example, cold weather can hinder the ionic conductivity in gel-based materials, reducing their effectiveness. Similarly, static electricity and dust accumulation can disrupt sensor readings, highlighting the need for improved material designs. Although ionic liquids offer some advantages, such as maintaining liquid form and high ionic conductivity even at low temperatures, they are not immune to challenges like dust and debris contamination. These issues require the development of materials that can maintain stable conductivity in diverse environmental conditions, integrate antistatic properties, and withstand physical contamination.

#### 6.1.6. Magnetic Tactile Sensors

Magnetic tactile sensors have become essential tools for detecting forces. These sensors use changes in the magnetic field to detect normal and shear forces and torque, ensuring flexibility, durability, and high sensitivity. Their adaptability makes them useful for applications that require precise force measurements in dynamic environments. The Pyramid Adapter Sensor incorporates contactless magnetic sensing into prosthetic devices, detecting forces up to 2500 *N* and torque up to 120 Nm. However, it only detects the axial force and the sagittal torque and requires calibration to address nonlinearities [143]. Textile sensors based on MXene include dual-mode proximity and pressure detection, providing scalability and flexibility for wearable devices, although their sensitivity decreases with bending and requires calibration for accuracy [145]. The ReSkin sensor uses magnetic microparticles and machine learning to achieve high temporal and spatial resolution in a cost-effective and replaceable design, but it is sensitive to magnetic fields, drifts over time, and lacks multipoint contact capability [144]. Split-type magnetic tactile sensors offer 3D force sensing with great durability and underwater functioning. However, they are susceptible to magnetic interference and tilt and twist inaccuracies [147].

Magnetic tactile sensors face challenges such as hysteresis caused by viscoelastic materials and vulnerability to external magnetic interference that compromise their reliability and accuracy. Although the FM-PLS sensor is encased in 3D printed PLA plastic to protect against dust and moisture, it is not suitable for extreme environmental conditions [146]. NdFeB magnets provide potential solutions by maintaining strong magnetic characteristics at low temperatures, enabling self-cleaning surfaces and static protection. However, NdFeB has certain drawbacks. It is fragile and can break under stress, is prone to corrosion in moist environments without protective coatings, and loses magnetic properties at high temperatures. Furthermore, its powerful magnetic fields can interfere with the near components, reducing the accuracy of the sensor array. Integrating NdFeB into tactile sensors is also complex and expensive, and its reliance on rare-earth elements raises environmental and sustainability concerns.

#### 6.1.7. Optical Tactile Sensors

Optical sensors have become more popular in tactile sensing due to their ability to detect mechanical inputs through changes in light patterns. These sensors use light-emitting diodes (LEDs), lasers, and fiber optic cables to measure pressure, texture, and deformation. Texture feedback increases object interaction and manipulation [152]. Fiber Bragg grating technology uses wavelength shifts to monitor mechanical strain and temperature changes with high accuracy [151]. Biomimetic sensors, such as TacTip and PapillArray, mimic the structure of human skin to improve robotic grip and texture detection [148]. TacTip uses an internal pin array to feel surfaces, whereas PapillArray uses silicon pillars to record forces and torques, allowing robots to safely grab a variety of things. Meanwhile, 2D tactile sensors embedded in soft silicone pads and equipped with deep learning algorithms detect accurate force and position [151]. These sensors combine precise spatial resolution and excellent accuracy with a simple, cost-effective design that promotes scaling. Organic optoelectronic sensors utilize multifunctional diodes that act as light emitters and detectors, resulting in flexible, large-area tactile arrays suitable for wearable applications [149].

The misalignment of optical components may decrease precision, resulting in lower sensitivity and inconsistent performance. Furthermore, environmental factors such as surface contaminants, varying lighting conditions, and extreme temperatures can lead to material degradation, especially in cold situations where static electricity buildup provides additional dangers. Silicone-based materials, which are commonly used in optical sensors, may struggle to maintain durability and precision under such conditions, and the complexity and cost of adding LEDs and lasers for accurate signal processing further limit their practical application. Addressing these challenges requires the development of more robust materials, such as hybrid combinations such as PDMS and elastic resin, which not only improve flexibility and durability, but also improve biomimetic designs for advanced applications such as robotic grasping.

#### 6.1.8. Vision-Based Tactile Sensors

Vision-based sensors have become more popular in tactile sensing due to their ability to detect mechanical inputs through changes in light patterns. These sensors use LEDs, lasers, and fiber optic cables to measure pressure, texture, and deformation. With advances in camera systems, marker configurations, and tunable performance parameters, vision-based tactile sensors offer versatility and precision compared to traditional tactile sensors. Marker-based vision sensing has emerged as a significant improvement in tactile sensing, allowing force measurement using calibrated markers such as single-layer, double-layer, color-coded, and optical flow designs. These sensors capture detailed texture and force interactions, making them useful in applications such as robotic manipulation and texture classification [156]. Systems such as DIGIT emphasize the importance of low-cost modular solutions that use tiny cameras and configurable elastomers to improve durability and scalability [154]. Event-based cameras improve sensors by offering higher temporal resolution and more efficient data processing than typical RGB cameras. Evetac detects shear pressures, slip events, and texture changes in real time, providing a sparse but highly informative output [157]. Additionally, techniques such as Depth from Defocus simplify hardware setups by using single-layer markers to achieve an accurate 3D force distribution with reduced complexity [153].

Vision-based tactile sensors face significant challenges that stem from the materials. These materials are particularly subject to environmental conditions, such as lighting variations, dust, and temperature fluctuations, which can affect sensor performance. Color-coded markers, which are often employed in these systems, are extremely sensitive to ambient light, whereas optical flow markers can be disrupted by shadows and reflections, resulting in inaccurate results. Cold weather further exacerbates these problems, as low temperatures can affect the performance of cameras by causing condensation on lenses, reducing image clarity, and impairing sensor responsiveness. Furthermore, prolonged exposure to cold environments can lead to hardware malfunctions, such as lens freezing, delayed image processing, and reduced battery efficiency, all of which can significantly hinder sensor reliability. Furthermore, in sensor design, there is an inherent trade-off between durability and sensitivity. Thicker elastomer layers improve robustness but reduce tactile resolution, whereas thinner layers increase sensitivity but are more susceptible to wear and deterioration. The lack of established criteria for force measurement limits performance evaluation across different sensors, and event-based sensors sometimes have difficulty reconstructing tactile data from a single measurement due to limited global configuration recovery.

**Table 4:** Comparison of advantages and disadvantages of tactile sensor technologies.

Sensor Type	Advantages	Disadvantages
Capacitive Tactile Sensors	High stretchability	Sensitive to noise
	High sensitivity over broad pressure or strain ranges	Performance saturation under high force or large strain
	Cost-effective fabrication methods	Potential missed or false detections for sharp/thin objects or at boundary regions
	Durability over repeated cycles	Susceptibility to moisture and contaminants (dust, sand, dirt)
Piezoresistive Tactile Sensors	High sensitivity over a wide pressure range	
	Mechanical flexibility	Hysteresis and viscoelastic effects can delay response or recovery
	Robustness and durability over repeted cycles	Susceptibility to moisture and contaminants (dust, sand, dirt)
	Low crosstalk and reliable signal acquisition when employing active-matrix or structured sensor designs	
Piezoelectric Tactile Sensors	High sensitivity for detecting subtle pressure changes	Complex fabrication processes
	Rapid response times suited for dynamic measurements	Degradation over extended use
	Flexible and conformable designs	Insufficient static pressure response, sensitive to dynamic forces
Triboelectric Tactile Sensors	Achieves high recognition accuracy	Exhibits baseline offset and drift in triboelectric signals
	High energy efficient	Lacks comprehensive protective measures against contaminants (dust, sand, dirt)
	Maintains flexible and stretchable structures	Requires multiple grasping cycles or repeated interactions to stabilize triboelectric signal amplitudes
	Demonstrates robust environmental resilience	
Electro-chemical Tactile Sensors	High sensitivity over a broad pressure range	Incomplete dissolution due to copper electrodes
	Ultra-low operating voltage	Vulnerability to high humidity
	Fast response times	Temperature sensitivity
	Integrated sensing and memory	Complex and expensive fabrication processes
Magnetic Tactile Sensors	High sensitivity and resolution for minimal forces	Susceptibility to Temperature and Alignment
	Capability to capture forces along multiple axes	Hysteresis effects in viscoelastic materials
	Silicone layers and textile-based designs provide soft or flexible exterior	Cross-talk among measurement axes, requiring careful calibration and modeling
		Magnetic sensing elements may be affected by nearby fields, bending, or film rotation.
Optical Tactile Sensors	High accuracy and resolution	Alignment and contact sensitivity
	Broad frequency or bandwidth capabilities	Z-axis force estimation can have notably higher errors compared to X and Y axes
	Silicone or elastomeric materials provides flexible and compliant structures	Accuracy degrades over time, requiring recalibration or model updates
		Possible wear and tear
Vision-based Tactile Sensors	High resolution and information (texture and deformation) capacity	Sensitivity to environmental factors (ambient light, reflections)
	Open-source designs and modular structures	Trade-off between durability and sensitivity
	Multimodal integration (tactile + visual data) for comprehensive force measurement and control	Bulky structure

### 6.2. Proprioceptive and Environmental Sensors

Beyond the tactile sensing capabilities that facilitate interactions with external objects, closed-loop prosthetic control extensively relies on sensors that monitor the internal states of the prosthesis and environmental conditions. Proprioceptive and environmental sensors are essential components in upper-limb prosthesis, as they provide continuous feedback that is required for real-time planning, stability, and adaptive limb motions. Internal prosthetic states such as joint angles, positions, and motion are monitored by proprioceptive sensors, which generally use joint angle encoders and inertial measurement units. These sensors enable precise kinematic control, allowing prosthetic limbs to mimic accurate and smooth movements similar to natural limb dynamics. Environmental sensors, such as integrated temperature sensors, monitor external factors that directly affect user comfort and prosthetic function. By integrating proprioceptive and environmental data into prosthetic control structures, these sensors improve the adaptability, reliability, and user comfort of the system, significantly improving the sophistication and simplicity of upper-limb prosthetic systems. This subsection discusses their operational principles, specific applications, and differences from tactile sensors, emphasizing their critical roles in closed-loop prosthetic control frameworks.

#### 6.2.1. Position (Proprioceptive) Sensors

Position sensors, such as absolute or incremental encoders, measure joint angles in prosthetic elbows, wrists, or fingers, providing crucial information for motion control. Accurate, low-latency kinematic feedback is essential in upper-limb prosthetics, where fine positional accuracy allows stable control loops and enables intuitive, integrated interaction for the user. In elbow modules, a shaft-mounted encoder provides angular position feedback for accurate flexion–extension control [158]. Potentiometer-based joint angle sensors embedded in selected thumb and finger joints provide continuous position feedback to the controller, enabling closed-loop position or force control of each motor in real time [159]. To reduce size while improving the linear angle feedback, the authors embed an infrared photodiode pair in the joint and let a variable thickness channel inside the link occlude the beam as the joint rotates, achieving high resolution with minimal electronics and no bulky optics [160]. A small Hall effect encoder has been used with both proportional derivative and biomimetic sliding mode controllers to provide reliable finger position feedback even when dynamically loaded [161]. The HIT-Hand has six distributed encoders, two on the thumb and one on each remaining finger, which provide joint-angle feedback to the impedance controller [162]. A 3D printed hand for transmetacarpal amputees uses Triaxis rotating Hall sensors to measure absolute joint angles, allowing closed-loop proportional derivative control [163]. The various implementations of encoders highlight that ongoing improvements in compact high-precision position sensing are essential to achieve the effective control required by advanced upper-limb prosthetics.

#### 6.2.2. Inertial Measurement Unit Sensors

The inertial measurement unit (IMU) is another important kinematic sensor for upper-limb prosthetics, providing precise orientation and motion data by integrating accelerometers, gyroscopes, and magnetometers into a compact structure suitable for wearable devices. Combining IMU signals on the forearm with surface EMG offers real-time limb orientation information that significantly improves pattern recognition accuracy, especially by compensating for variations in the arm position even when only one or two EMG channels are available [8,164]. The authors in [165] placed three high-precision IMUs on the upper arm, forearm, and hand, using the resulting segment orientation streams to build a motion unit classifier that recognized the daily tasks of the upper limb with high accuracy. Using a single wrist-mounted IMU and a random forest classifier, the authors were able to distinguish functional from non-functional movements of the prosthetic limb during everyday tasks [166]. Using only three IMUs and a relative angle and orientation algorithm, the authors showed that real-time joint angle feedback for the prosthesis can be performed without any external optical tracking system, providing a fully self-contained control loop [167]. Furthermore, IMUs offer temporally dense shoulder and elbow motion trajectories that can be used as the basis for training and validating EMG or MMG-based motion estimators, providing a non-invasive alternative to laboratory-based optical systems [168,169]. Among the calibration methods evaluated, manual alignment is accurate and user friendly to estimate elbow angles, leading the authors to recommend it as the preferred approach in clinical rehabilitation [170]. Finally, by embedding a 16 sensor textile pressure array and IMU in the prosthetic socket, the study synchronously mapped interface pressures and limb orientation during weighted grasp tasks [171]. In conclusion, the studies reviewed indicated that compact body-mounted IMUs are essential for AI-based classification and kinematic control of upper-limb prostheses.

#### 6.2.3. Temperature Sensors

Temperature sensors integrated into upper-limb prostheses detect object, ambient, and skin temperatures, giving users real-time thermal feedback that improves functional safety, embodiment, and task precision for close-loop control [172]. By recording EMG data alongside the LM35 sensor data, which are chamber and ambient temperatures, the study shows that temperature shifts modulate the amplitude of the EMG, suggesting that future prosthesis control algorithms should include temperature compensation [173]. The authors in [174] integrated a positive temperature coefficient (PTC) in a fully 3D printed prosthetic hand, achieving selective temperature sensing with very low crosstalk to strain and using only simple resistive read-out electronics. An ultrathin stretchable thermosistor that can accurately track skin-level temperatures and can be placed on prosthetics to provide rapid spatially resolved temperature feedback was presented in [175]. The transradial prosthesis integrates LM35 temperature sensors and force sensors in each fingertip to provide real-time grip temperature and pressure on its onboard touchscreen [176]. Beyond the hands, the thermistors built into a wearable orthosis continuously tracked the skin temperature of the upper limb to detect irritation and guide fit adjustments [177]. A dual-modality feedback interface combined a K-type thermocouple and a force sensor to deliver simultaneous temperature and force-based stiffness cues while compensating for temperature-induced force perception drift [178]. Temperature sensors embedded in upper-limb prostheses provide real-time thermal feedback that protects the device from hazardous temperatures and stabilizes control by compensating for temperature-induced signal drift.

Table 5 presents a comprehensive overview of the feedback modalities associated with various sensor types used in upper-limb prosthetic systems, based on the papers reviewed in this study. Tactile sensors collectively address the multifaceted sensory requirements necessary to replicate the tactile and proprioceptive functions of natural limbs. Proprioceptive sensors play a critical role in tracking the position of the limb and the dynamics of movement, which are essential to achieve intuitive control. This chapter underscores the necessity of integrating comprehensive sensory feedback mechanisms into upper-limb prosthetic systems to ensure natural, coordinated movements. However, their successful implementation depends on careful sensor selection, precise placement, and rigorous calibration to accommodate environmental variability, mechanical stresses, and the unique requirements of individual users. A comprehensive approach to sensor integration ensures that the prosthesis can adaptively respond to a wide range of tasks, thus enhancing functionality, comfort, and user satisfaction. Although sensor technologies are vital to providing meaningful feedback, it is equally important to understand that the performance of these sensors is closely related to the materials from which they are constructed. This highlights the need to carefully consider material selection not only for sensors themselves but also for the overall prosthetic design, balancing strength, comfort, and functionality to best meet the needs of users. In this section, the focus is on two practical design choices: the number of sensor types you include and how many channels each type uses. A single device, for instance, one inertial unit on the wrist or one EMG electrode, can manage simple open–close actions, but its signal shifts when the arm moves and tells us little about the wider context. Adding just one more sensor, typically pairing an EMG with an IMU, already makes intent detection far more reliable, although it does mean a bit more work to fuse and tune the signals. If we stick to only a handful of channels, the wiring, data flow, and power remain modest. At the other extreme, high-density EMG sleeves with around 128 electrodes can identify more than 30 hand gestures with excellent accuracy, yet they generate so much data that some form of onboard compression or processing is essential.

## 7. Material Selection in Upper-Limb Prosthetics: Balancing Strength, Comfort, and Function

Material selection is a critical aspect of upper-limb prosthetic design since the choice of material directly affects strength, durability, weight, flexibility, and biocompatibility. Table 6 groups these materials into six categories—structural and frame materials, soft interface materials, socket materials, functional component materials, cosmetic covers, and sensor and electronic materials—each tailored to a specific role in prosthesis performance and wearer comfort.

### 7.1. Structural and Frame Materials

The structural framework of upper-limb prosthetics relies on materials that offer an optimal balance of strength, weight, and durability, ensuring both functionality and user comfort. Aluminum is frequently employed due to its low density, resistance to corrosion, and relatively high mechanical strength, making it a versatile choice for many prosthetic components [179,180]. Titanium, known for its excellent strength-to-weight ratio and biocompatibility, is especially suitable in areas subject to high stress or load-bearing, thereby contributing to the longevity of the prosthesis [181]. Stainless steel, while heavier than aluminum or titanium, offers robust mechanical performance and cost effectiveness, which can be advantageous in situations where budget constraints or specific mechanical requirements must be met [182,183]. Carbon fiber has emerged as a leading choice in cutting-edge prosthetic design due to its remarkable tensile strength, impact resistance, and lightweight properties, which enable enhanced user mobility and comfort [184,185]. Collectively, these materials form the backbone of upper-limb prostheses, aligning structural stability with ergonomics and patient-specific needs.

### 7.2. Soft Interface Materials

Soft interface materials are critical in ensuring optimal comfort, load distribution, and skin health within upper-limb prosthetic systems. Silicone, a popular choice due to its biocompatibility and ease of cleaning, effectively mimics the mechanical properties of soft tissue and provides a snug, low-friction fit for residual limbs [186]. Thermoplastic elastomers (TPEs), such as styrene-ethylene-butylene-styrene (SEBS), offer adjustable hardness and flexibility, making them ideal for creating customized interfaces that accommodate user-specific needs [187,188]. Gel liners, commonly composed of medical-grade silicone or mineral oil-based polymers, further enhance wearer comfort by conforming closely to limb contours, mitigating shear forces, and reducing the risk of skin irritation [189,190,191]. As a result, the thoughtful selection and integration of soft interface materials can significantly influence prosthetic comfort and user satisfaction, particularly for devices intended for long-term daily use.

### 7.3. Socket Materials

Socket materials play a vital role in upper-limb prosthetic design, as they serve as the primary interface between the prosthesis and the residual limb. Acrylic resin, widely appreciated for its strength and ease of lamination, creates rigid yet customizable sockets that can accommodate reinforcement materials, allowing for tailored stiffness and support [192]. Polypropylene, a lightweight and thermoplastic option, is well-suited for both diagnostic and definitive sockets because it can be reheated and remolded, enabling prosthetists to make precise adjustments for optimal fit and comfort [193]. Polyethylene, another commonly used thermoplastic, offers enhanced flexibility and impact resistance; it is particularly beneficial for patients requiring a more adaptable socket design that can better accommodate fluctuations in limb volume [194]. Overall, choosing the right socket material is essential for ensuring proper load distribution, stability, and wearer comfort, underscoring the importance of material selection in contemporary prosthetic practice.

### 7.4. Functional Components Materials

Functional components in upper-limb prosthetics—encompassing joints, connectors, and moving parts—require materials that seamlessly blend mechanical strength, durability, and weight efficiency. Steel alloys, for instance, have long been lauded for their robust performance and resistance to wear, especially in high-stress areas such as hinges and load-bearing pivots [195]. Polycarbonate, prized for its impressive impact resistance and transparency, can be molded into complex shapes while maintaining a lightweight form, making it an ideal choice for prosthetic hand casings and protective covers [196]. In addition, advanced polymers—ranging from reinforced nylon blends to high-performance thermoplastic composites—have become increasingly prevalent due to their favorable strength-to-weight ratios and low-friction coefficients; these properties facilitate smoother joint articulation and reduce user fatigue [10]. Through careful selection and optimization of these materials, engineers and clinicians can significantly enhance functionality, extend the device’s service life, and improve overall user satisfaction in upper-limb prostheses.

### 7.5. Cosmetic Covers

Cosmetic materials are pivotal in upper-limb prosthetics, helping to merge functionality with a natural aesthetic that bolsters user confidence and satisfaction. Foamed polyurethane is particularly valued for its lightweight composition and malleability, allowing it to be sculpted to mimic the contours and softness of natural tissue while providing a protective layer over the internal components [197]. In contrast, polyvinyl chloride (PVC) or silicone skins serve as outer coverings designed to replicate the look and feel of human skin, offering a wide range of pigmentation options and surface textures. These skins not only deliver an appearance closer to that of a biological limb but also help protect the prosthesis from environmental factors such as moisture and abrasion [198,199]. By thoughtfully selecting and customizing these materials, prosthetists can create cosmetic solutions that are durable, comfortable, and visually appealing, thus enhancing overall prosthetic performance and user acceptance.

### 7.6. Sensors and Electronics Materials (For Myoelectric Prosthetics)

Sensors and electronics are central to modern upper-limb prosthetics, enabling advanced functionalities such as myoelectric control, haptic feedback, and wireless connectivity. Conductive materials like copper and silver are commonly employed in wiring, electrodes, and circuit boards due to their high electrical conductivity and reliable signal transmission, which is critical for interpreting and relaying bio-signals [200,201,202]. Lithium-ion batteries, characterized by their superior energy density and longer life cycle, provide a stable and lightweight power source for sophisticated prosthetic systems, supporting motors, sensors, and communication modules without imposing excessive weight on the user [203]. Enclosures for these electronic components often utilize robust plastic or metal alloys, chosen for their durability, impact resistance, and ease of manufacturing, ultimately safeguarding sensitive circuitry from environmental factors such as moisture, dust, and physical shocks. By integrating these carefully selected materials, developers and clinicians can deliver cutting-edge prosthetic devices that offer both reliable performance and user-friendly features.

## 8. Discussion

The reviewed literature underscores the rapid evolution of EMG-based control strategies for upper-limb prostheses, revealing noteworthy strides in signal acquisition, feature extraction, and ML integration [9,13,14]. Traditional models such as support vector machines, k-Nearest Neighbors (kNN), and ANNs have consistently delivered reliable classification accuracies upward of 90%, owing in part to well-established preprocessing methods like band-pass filtering and notch filtering [9]. However, more sophisticated deep learning approaches, such as CNNs, further improve robustness and user functionality when confronting real-world challenges—specifically, noise contamination, electrode displacement, and inter-subject variability [15,18].

Several pivotal themes emerge. First, incremental and adaptive learning methods address day-to-day variability and electrode shifts. For instance, CIIL leverages environmental feedback to sustain system reliability over extended use [16], while DANN frameworks tackle semi-supervised scenarios by augmenting data synthesis for real-time adaptability [29]. These techniques seek to reduce the recalibration burden, thus improving user convenience and experience [20].

Second, research converges on the significance of resilient hardware and open-source solutions that enhance affordability. Efforts such as HMG, magnetic detection for muscle contractions, show potential in noisy or resource-limited environments [27], and cost-effective, 3D-printing-based prostheses further broaden accessibility [31,49]. With the introduction of user-friendly frameworks, for example, the SDU simplifies calibration and increases adoption rates among both clinicians and patients [24].

Third is hybrid control and multimodal feedback as key developments for refined prosthetic operation. Fusing EMG signals with steady-state visual evoked potentials (SSVEPs), optical sensors, or tongue-based interfaces not only improves classification under fatigue but also shortens task completion times [22,47]. Meanwhile, haptic or proprioceptive feedback systems, such as real-time grip state feedback devices, demonstrate superior performance by giving users continuous cues on posture or grip force [43,44].

Finally, continuous progress in XAI and advanced ML highlights interpretability and generalization as paramount. XAI methods shed light on feature relevance and electrode placement, reducing computational complexity and enhancing universality [19]. Transfer learning paradigms, such as those that repurpose CNNs trained on large datasets to new prosthetic control tasks, help manage inter-subject variability and expedite model training [17,39].

Collectively, these innovations illustrate an integrated trajectory toward more robust, adaptive, and patient-centered prosthetic systems. From improving domain adaptation strategies and minimizing user recalibration times to optimizing hardware solutions for cost and accessibility, researchers are navigating a balanced path between high performance and everyday practicality [25,26,35]. Although challenges such as daily EMG variability and multi-degree-of-freedom control persist, ongoing developments in reinforcement learning, transfer learning, and low-cost sensor technologies promise further transformations, bringing us closer to prosthetics that empower individuals with limb differences to regain high levels of independence and quality of life [34,37,46].

Overall, the advancements synthesized in this review strongly indicate that the future of EMG-based prosthetic technology lies in the deep integration of adaptive algorithms, multimodal feedback, and economically viable design principles. Future research must concentrate on large-scale, longitudinal studies that validate these methods under real-world conditions, ensuring their reliability, safety, and user acceptance for broad clinical deployment [5,48,54,55]. In addition to clinical validation, the real-world usability of prosthetic devices depends heavily on their performance under varying environmental conditions. Weather-related factors such as low temperatures, humidity, and airborne dust can alter the behavior of sensors and materials, potentially affecting reliability. For example, capacitive sensors may lose precision due to surface contamination, while cold temperatures can cause stiffening in certain polymer-based sensing elements. These limitations underscore the importance of incorporating weather-tolerant design features—such as protective coatings, hydrophobic materials, and thermally stable polymers—into prosthetic systems.

A future improvement in this regard could be the development of adaptive or fast calibration-based control, where conventional machine learning algorithms are implemented with automatic feature extraction, adapting these features to the target subject, thus becoming light and high performing, solving feature variability.

Upper-limb prosthetic devices rely on carefully selected materials to balance strength, weight, comfort, and biocompatibility across multiple functional areas. Structurally, aluminum, titanium, stainless steel, and carbon fiber form durable frames that withstand daily stresses without sacrificing wearer comfort. Soft interface materials such as silicone, thermoplastic elastomers, and gel liners help distribute loads evenly and protect skin integrity, improving user comfort and reducing irritation. Socket materials—including acrylic resin, polypropylene, and polyethylene—support a well-fitted interface between the prosthesis and the residual limb, offering rigidity or flexibility as needed. In terms of functional components, steel alloys and advanced polymers provide robust mechanical performance and smooth articulation in joints, connectors, and other moving parts. For aesthetics and user confidence, cosmetic covers made of foamed polyurethane, PVC, or silicone replicate the natural look and feel of human skin. Meanwhile, sensors and electronics components (e.g., copper or silver wiring, and lithium-ion batteries) enable advanced functionalities such as myoelectric control and haptic feedback. Despite these advances, several unresolved research challenges still exist. First, the mechanical mismatch between synthetic socket materials and biological tissue can lead to discomfort, pressure sores, or long-term wear issues, especially during extended daily use. Second, the development of adaptive or smart materials that respond dynamically to movement or temperature remains limited, impeding user comfort in varying environments. Third, ensuring the long-term durability and biocompatibility of materials—particularly those exposed to sweat, temperature fluctuation, or mechanical stress—continues to pose challenges. Fourth, integrating rigid electronic components (e.g., sensors, wiring, and batteries) with soft materials without compromising structural integrity or increasing bulk is a persistent constraint. Finally, there is a pressing need for personalized prosthetic materials that can be tailored to individual anatomical and functional demands, which requires advancements in additive manufacturing and multi-material design. Altogether, selecting and integrating these distinct material categories is pivotal for optimizing prosthetic performance, longevity, and user satisfaction. Future research should prioritize multi-material systems that combine structural strength (e.g., carbon fiber) with soft, adaptive components (e.g., hydrogels). Bioinspired materials with gradient stiffness and smart polymers with shape-memory effects offer promise for enhanced comfort and responsiveness. Focus should also be given to durability, environmental resistance, and sustainable, recyclable materials.

Based on the literature review in the period of 2018–2025, the tendon-based actuation mechanism remains the most popular and widely used mechanism type among other underactuated mechanisms. This dominance is potentially due to its simple structure and effectiveness in the controlling of parts of a robotic hand. It is revealed that the linkage-based actuation is usually used in prosthetic solution with rigid structure, whereas the tendon-based type could be used in both rigid or soft robotics applications. The studies that used a pneumatic actuation mostly proposed the design of a soft robotic hand or finger. The main focuses of the surveyed works were to design a versatile robotic hand with high dexterity and grasping capabilities, having a lightweight body. However, there is still a trade-off between designing of a robotic hand with such requirements and its practical applications. Although some of the works could achieve their goal in terms of adaptive grasping and dexterity, their proposed hands suffer with bulky design or heavy weight [76,82,87,91,92]. Pneumatic actuation is an attractive mechanism due to its high performance in adaptability and grasping objects with different shapes, and reduced number of actuators. However, issues with the generation of the source of actuation (air pressure) still remain as open questions since it might add additional weight for the prosthetic hand system and require complex maintenance during exploitation. The linkage-based actuation can be a solution in optimizing the number of actuators used in rigid mechanisms. A combination of various type of linkage-based mechanisms can be used to provide more degrees of freedom while using less number of motors as in [87]. Moreover, control challenges remain an active area of research due to the nonlinearities and complexities of coupled drive mechanisms. In tendon-driven mechanisms, force is transmitted indirectly from remotely located actuators via tendons, introducing challenges associated with nonlinearities such as friction, hysteresis, and tendon slack. Additionally, joint angles cannot always be accurately inferred from actuator positions alone, making the integration of dedicated sensors essential for achieving precise and reliable control. For example, force or tension sensors can enable a moderate level of control; however, achieving high precision typically requires accurate dynamic modeling to compensate for system nonlinearities and uncertainties. Alternatively, linkage-driven mechanisms offer deterministic and repeatable kinematics, making them well-suited for precise sensing applications. Unlike tendon-driven systems, joint angles in linkage-based designs are directly measurable, allowing for seamless integration with IMU sensors and enabling accurate multi-DOF tracking. Linkage-driven mechanisms, due to their structural rigidity, are well-suited for sensor integration. Nevertheless, accommodating these sensors often necessitates careful alignment and additional space, which may compromise the compactness and lightweight nature desired in prosthetic designs. Although linkage-driven mechanisms are advantageous in achieving precise multi-DOF control, they lack the adaptability and compliance that tendon-driven systems can offer. Due to their reliance on compressed air for actuation, pneumatic mechanisms are inherently nonlinear and difficult to model, complicating the implementation of precise control strategies. In contrast to tendon- and linkage-driven systems, their limited sensor compatibility further restricts their suitability for accurate multi-DOF control.

Capacitive sensors provide high spatial resolution, low power consumption, and high sensitivity, making them suitable for precise force control. In contrast, piezoresistive sensors operate effectively in dynamic situations and allow cost-effective fabrication, making them suitable for large-scale deployment. Although piezoelectric sensors have a high frequency response, their limited spatial resolution and susceptibility to mechanical stress can make them unsuitable for long-term applications. Triboelectric sensors, while energy efficient, can suffer signal noise in dusty environments. Electrochemical sensors have ultra-low operating voltage and multifunctionality; however, they are susceptible to temperature variations and contamination. Magnetic sensors provide enhanced multiaxis force detection but are prone to interference from external fields or hysteresis, which reduces their accuracy in severe situations. Optical sensors provide high precision and broad bandwidth capabilities, but require precise alignment and can degrade in dusty or cold environments, necessitating regular maintenance. Finally, while vision-based systems offer greater texture analysis and high-resolution feedback, they remain complex and cost prohibitive for widespread prosthetic use.

Capacitive and piezoresistive tactile sensors present an optimal option for integration into upper-limb prosthetic systems [204,205,206,207]. In adverse weather environments, such as Kazakhstan, the durability of the sensor and the stable characteristics of the material are essential. Dust gathering on sensor surfaces can affect the accuracy of capacitive sensors, whereas hysteresis or lessened sensitivity can occur in piezoresistive and piezoelectric materials due to stiffening at lower temperatures. Techniques such as protective coatings, conductive fillers, and biocompatible materials can help prevent these problems while also improving user comfort and safety. In practical applications, piezoresistive sensors are more robust in cost-sensitive, high-movement activities, whereas capacitive sensors outperform when high resolution is required and can be protected against environmental contaminants. A potential advancement can be the use of hybrid sensor systems that combine capacitive, piezoresistive, or piezoelectric elements with biodegradable or self-healing elastomers to develop multipurpose, biocompatible solutions. Next-generation prostheses that provide robust and stable tactile feedback even in adverse environmental circumstances can be developed with the intrinsic characteristics of each sensor and using design techniques to address environmental constraints. Since existing modalities like force, slip, temperature, and position are well represented in the literature, future developments could focus on incorporating additional sensory capabilities, such as texture recognition and vibration frequency sensitivity, that have significance for surface discrimination and precise manipulation tasks. Improving tactile feedback in this direction will contribute to the biomimetic performance of prosthetic and robotic systems, enabling more natural and accurate interactions with complex environments.

## 9. Conclusions

This review has provided an in-depth analysis of the key advancements in upper-limb prostheses, focusing on control strategies, sensor integration, transmission mechanisms, and material innovations. A systematic search was conducted across multiple databases to examine how machine learning, advanced control methodologies, and novel materials contribute to the development of more responsive and functional prosthetic devices.

The study explored EMG-based control strategies, highlighting their evolution from traditional PID controllers to more sophisticated approaches like fuzzy logic and deep learning models. These techniques enhance real-time adaptability and improve user experience. Additionally, tactile sensing technologies, including capacitive and piezoresistive sensors, were reviewed for their role in providing sensory feedback, improving grip stability, and enhancing object manipulation. The mechanical transmission mechanisms utilized in prosthetic designs such as tendon-driven, linkage-based, and pneumatic actuators were compared in terms of efficiency, durability, and biomechanical compatibility. Furthermore, the impact of new materials and socket designs on user comfort, safety, and long-term usability was assessed, with particular emphasis on lightweight composites and customizable structures enabled by additive manufacturing.

Despite these advancements, several challenges remain. The variability of EMG signals and the sensitivity of electrode placement continue to affect control accuracy. The weight and complexity of actuation mechanisms present trade-offs between robustness and ease of use. Additionally, the durability of sensors and materials in diverse environmental conditions remains a concern. Future research should focus on large-scale user trials, the integration of adaptive learning algorithms, and the development of cost-effective fabrication methods, such as 3D printing, to enhance accessibility and affordability. By addressing these challenges, the field of prosthetics can continue progressing toward devices that not only restore lost functionality but also offer a seamless and intuitive user experience for individuals with upper-limb loss.

## Figures and Tables

**Table 1:** Machine learning methods in upper-limb prosthesis.

Machine Learning Methods	Sensors Used	Subject Types	Accuracy/Key Results	Implementation
SVM, k-NN, ANN	Surface EMG	Amputees, Non-amputees	Up to 97% [9,13]	Classification for dataset; hand-gesture classification
CNN (Transfer Learning)	sEMG signals	Healthy subjects	95.45% [14]	Real-time classification
CNN + SVM (Time–Frequency Features)	sEMG signals	Healthy subjects	99.9% [18]	Hybrid learning
ResNet (Residual Learning)	sEMG signals	Primarily amputees (lower-limb focus, adaptable to upper limb)	95.34% [45]	Deep learning
Temporal Multi-Channel Transformers	Surface EMG	Healthy subjects, Non-amputees	Up to 95% [15]	Deep learning
XAI Approach	Surface EMG	Amputees	93.11%; high interpretability [19]	Explainable AI
k-NN + Advanced Filtering (Wiener, Hampel)	Surface EMG	Transhumeral amputees	Up to 88% [20]	Supervised learning
Transient EMG Classifier	sEMG signals	Amputees, Non-amputees	High accuracy for cross-subject recognition [23]	Supervised learning
Hybrid Neural Network + Transfer Learning	EMG signals	Elderly population (fall-risk gait)	95% [17]	Hybrid learning
Dual Multi-Classifier (Fuzzy Logic)	EMG signals	Not specified	High reliability in noisy conditions [25]	Fuzzy logic
Optimized PR System for *Hannes* Prosthesis	sEMG signals	Amputees	F1 score 99.8% [35]	Pattern recognition
Reinforcement Learning-Based Personalization	EMG signals	Not specified	High kinematic-estimation accuracy [46]	Reinforcement learning
MDSDA Network (Domain Adaptation)	sEMG signals	Healthy (configurable for broader use)	Improved robustness across domain shifts [39]	Transfer learning
Hybrid Tongue–Myoelectric Interface	EMG + Tongue sensor	Not specified (in-lab tests)	19% improvement in task times [47]	Hybrid learning

**Table 2:** Comparison of actuation mechanisms presented in the literature from 2018 to 2025.

Actuation Mechanism	Source of Actuation	Advantages	Disadvantages	Robotic Hand/Robotic Gripper/Robotic Finger	Application Type	Refs.
Tendon-driven	Electric motor	Independent control of stiffness and position, versatile motion	Bulky design, heavy weight	Robotic hand	Rigid robotics	[76]
	Electric motor	Wrench estimation	Complex structure	Robotic finger	Rigid robotics	[77]
	Thermal activation	Attractive design, natural size, good dexterity	Only lightweight objects can be grasped and lifted	Robotic hand	Rigid robotics	[78]
	Electric motor and fluid pressure	Attractive look, good bending angle and force	Complex design of actuation, bulky size, heavy weight, no feedback control	Robotic hand	Soft robotics	[79]
	Tendon	Various shapes of objects for grasping, simple scheme of actuation, fast response	Ugly design	Robotic gripper	Soft robotics	[98]
	Electric motor	Design similar to human finger, simple actuation scheme	Complex structure, development of the design can be costly	Robotic finger	Rigid robotics	[80]
	Electric motor	Design close to natural, effective swing mechanics, high impact resistance, increased swing speed	Tactile sensors are not integrated, limited grasping motions of the fingers	Robotic hand	Rigid robotics	[81]
	Tendon	Attractive design, lightweight, tendon routing is not complex, fast response	Stiffness is hight dependent on string properties, might require frequent maintenance due to multiple strings in the design	Robotic finger	Rigid robotics	[99]
	Electric motor and fluid pressure	Natural appearance of the design, good grasping and lifting performance	Complicated source of actuation, too bulky design	Robotic hand	Soft robotics	[82]
Tendon-driven	Electric motor	Good dexterity, attractive fashion, multiple grips can be achieved, lightweight	Feedback control is not integrated, design material could be improved	Robotic hand	Rigid robotics	[83]
	Electric motor	Antagonistic actuation, fast response of fingers, good grasping performance	Not natural appearance, heavy weight	Robotic hand	Rigid robotics	[84]
	Electric motor	Elongatable fingers, high dexterity, feedback control based on soft tactile sensors, improved grasping performance	Might require complex maintenance due to complex actuation structure	Robotic finger	Rigid robotics	[85]
Linkage driven	Electric motor	Good grasping, reduced number of motors, strong load bearing	Bulky design with unnatural appearance, only three fingers	Robotic hand	Rigid robotics	[86]
	Electric motor	Only single actuator, can grasp objects with various shapes, acceptable hand closing time	Bulky design and heavy weight, absence of feedback control	Robotic hand	Rigid robotics	[87]
	Electric motor	Acceptable grasping performance, load lifting force	Bulky and rude design, only three fingers, complex structure with several driving modules	Robotic hand	Rigid robotics	[88]
	Electric motor	Simple structure, acceptable adaptability for grasping different shapes	Unnatural appearance, no feedback	Robotic gripper	Rigid robotics	[100]
	Electric motor	Can accomplish 33 postures in the GRASP taxonomy	Cables cannot bear high loads, bulky design	Robotic hand	Rigid robotics	[69]
Pneumatic	Air pressure	Grasping different shapes, simple actuation scheme, fast response	Unnatural appearance, bulky design, only four fingers, requires pressure source for actuation	Robotic hand	Soft robotics	[91]
	Air pressure	Fast response, good grasping and pinching	Requires pressure source	Robotic gripper	Soft robotics	[92]
	Air pressure	Lightweight, fast grasping response, good grasping, good stiffness	Complicated scheme of actuation, the repair might be costly	Robotic hand	Rigid/Soft robotics	[93]
	Air pressure	Can grasp different shapes	Unnatural design, low grasping force	Robotic hand	Soft robotics	[94]
	Hydrogen peroxide	Lightweight, compact size, good grasping	Complex actuation scheme, requires peroxide solution pack, no feedback control	Robotic hand	Soft robotics	[95]
	Air pressure	Simple actuation scheme	Unnatural appearance, low grasping and payload force	Robotic gripper	Soft robotics	[96]
	Air pressure	Good grasping performance, feedback control	Unnatural view, only four fingers, complex design	Robotic hand	Soft robotics	[97]

**Table 5:** Feedback modalities across sensor types.

Sensor Type	Feedback Modality
Capacitive Tactile Sensors	Force
Piezoresistive Tactile Sensors	Force, Slip
Piezoelectric Tactile Sensors	Force, Slip
Triboelectric Tactile Sensors	Force, Slip
Electro-chemical Tactile Sensors	Force, Temperature
Magnetic Tactile Sensors	Force
Optical Tactile Sensors	Force, Slip, Position
Vision-based Tactile Sensors	Force, Slip
Position (Proprioceptive) Sensors	Position/Angle
IMU Sensors	Orientation, Acceleration, Angular velocity
Temperature Sensors	Temperature

**Table 6:** Summary of materials used in upper-limb prosthetics, including their key properties and common applications.

Material	Key Properties	Common Uses
Aluminum	Lightweight, Corrosion-resistant	Mechanical components
Titanium	Lightweight, Strong, Durable	High-performance or premium prosthetics
Acrylic resin	Durable, customizable	Forming rigid sockets
Polypropylene	Lightweight, easily moldable	Sockets, flexible components
Polyethylene	Flexible, durable	Sockets (in some cases)
Silicone	Flexible, durable, skin-friendly	Prosthetic liners
Thermoplastic elastomers (TPEs)	Soft, stretchable, lightweight	Liners or soft sockets
Gel liners	Provide cushioning, reduce friction	Between the skin and prosthetic socket
Stainless steel	Strong, durable, heavier	Areas where high strength is critical
Carbon fiber	Lightweight, strong, highly durable	Advanced prosthetics (excellent strength-to-weight ratio)
Steel alloys	Robust, suitable for high-stress applications	Hinges, locking mechanisms
Polycarbonate	Lightweight, strong	Structural or cosmetic components
Advanced polymers (e.g., DELRIN)	Stable, wear-resistant, suitable for small precision parts	Bushings, small moving parts
Copper and silver	High electrical conductivity	Wiring, conductive electrodes
Lithium-ion batteries	High energy density, stable power supply	Power source for motors, sensors
Plastic and metal alloys	Protective, durable, can be molded or machined	Housing microprocessors and electronic components

## Data Availability

Not applicable.
